# Intrinsic D614G and P681R/H mutations in SARS-CoV-2 VoCs Alpha, Delta, Omicron and viruses with D614G plus key signature mutations in spike protein alters fusogenicity and infectivity

**DOI:** 10.1007/s00430-022-00760-7

**Published:** 2022-12-30

**Authors:** Ritika Khatri, Gazala Siddqui, Srikanth Sadhu, Vikas Maithil, Preeti Vishwakarma, Bharat Lohiya, Abhishek Goswami, Shubbir Ahmed, Amit Awasthi, Sweety Samal

**Affiliations:** 1 Translational Health Science and Technology Institute, NCR Biotech Science Cluster, Faridabad, Haryana 121001 India; 2 Immunobiology and Immunology Core Laboratory, Translational Health Science and Technology Institute, NCR Biotech Science Cluster, Faridabad, Haryana 121001 India; 3 Centralized Core Research Facility (CCRF), All India Institute of Medical Science (AIIMS), Delhi, India

**Keywords:** SARS-CoV-2 variants of concern, Omicron, Delta, Infectivity, Virus entry

## Abstract

The SARS-CoV-2 virus has been rapidly evolving over the time and the genetic variation has led to the generation of Variants of Concerns (VoC), which have shown increased fitness. These VoC viruses contain the key mutations in the spike protein which have allowed better survival and evasion of host defense mechanisms. The D614G mutation in the spike domain is found in the majority of VoC; additionally, the P681R/H mutation at the S1/S2 furin cleavage site junction is also found to be highly conserved in major VoCs; Alpha, Delta, Omicron, and its’ current variants. The impact of these genetic alterations of the SARS-CoV-2 VoCs on the host cell entry, transmissibility, and infectivity has not been clearly identified. In our study, Delta and D614G + P681R synthetic double mutant pseudoviruses showed a significant increase in the cell entry, cell-to-cell fusion and infectivity. In contrast, the Omicron and P681H synthetic single mutant pseudoviruses showed TMPRSS2 independent cell entry, less fusion and infectivity as compared to Delta and D614G + P681R double mutants. Addition of exogenous trypsin further enhanced fusion in Delta viruses as compared to Omicron. Furthermore, Delta viruses showed susceptibility to both E64d and Camostat mesylate inhibitors suggesting, that the Delta virus could exploit both endosomal and TMPRSS2 dependent entry pathways as compared to the Omicron virus. Taken together, these results indicate that the D614G and P681R/H mutations in the spike protein are pivotal which might be favoring the VoC replication in different host compartments, and thus allowing a balance of mutation vs selection for better long-term adaptation.

**Supplementary Information:**

The online version contains supplementary material available at 10.1007/s00430-022-00760-7.

## Introduction

Severe Acute Respiratory Coronavirus 2 (SARS-CoV-2) is a positive-sense, single-stranded, enveloped RNA virus which belongs to the Coronaviridae family, genus beta corona virus [1]. Since the emergence of the SARS-CoV-2 pandemic in late 2019, the virus has been evolved and SARS-CoV-2 Variants of Concerns (VoCs) have emerged. A few VoC viruses were highly infectious and transmissible and resulted in causing the second (Alpha variant) and third pandemic waves (Delta variant) [2]. In November 2021, another important VoC named as Omicron emerged in South Africa, and rapidly spread to many parts of the world, resulted in the fourth pandemic wave, the Omicron virus was classified as the fifth VoC (COVID19.who.int) Omicron viruses are further evolving and currently there are several lineages; BA.1, BA.1.1, BA.2, BA.3, BA.4 and BA.5 variants which are causing current infections in the population [3–5].

The first important variant was observed in early 2020 [6], which consists of a single aspartic acid at 614 position to glycine mutation (D614G) in the spike protein, and in time, this mutation proved to be highly infectious and adventitious for SARS-CoV-2 virus fitness [7]. D614G virus mutation eventuated in a higher viral load in the upper respiratory tract, although the disease severity was not significantly altered in compared to the Wuhan-1 strain [8]. Later, the Alpha variant (B.1.1.7) was identified in the United Kingdom in September 2020 and declared as a variant of concern (COVID19.who.int). The Alpha variant consists of 17 mutations in the spike protein [9], and the major RBD mutation N501Y has been shown enhanced binding to ACE2 and resulted in escape neutralization [10]. The Alpha variant also contains D614G and a cleavage site mutation P681H [11, 12]. The third major pandemic wave was caused by the Delta variant (B.1.617.2) in late 2020 in India and afterwards spread all over the world in more than 90 countries [13]. The Delta variant infection results in severe disease [14, 15], high replication in the lower airway tract, enhanced syncytial formation, significant increase in the virus entry and a marked reduction in vaccine effectiveness [16–18]. The Delta variant contains D614G and the P681R mutations. Currently, the Omicron variant and it’s lineages (BA.1, BA.2, BA.3, BA.4, BA.5) are the dominant VoC which are circulating in population. The Omicron and it’s lineages contain > 30 mutations in the spike protein and also harbors the D614G and P681H mutations [19, 20]. The Omicron variant lineages now dominate the majority of clinical cases and the number of cases against BA.4 and BA.5 are rising [5, 20]. The Omicron virus and its variants’ manifest high transmissibility with milder symptoms [21]. However, the Omicron VoC has been shown to be more infectious and has been evading the immune responses to vaccines [22, 23].

SARS-CoV-2 virus mediates the infection by binding of the envelope spike protein (S) to the cellular receptor hACE2, which is highly expressed on epithelial cells of the airway tract, lung and intestine [24, 25]. The binding to the host receptor facilitates conformational changes leading to the fusion of virus and host cell membrane. The spike protein is expressed as an inactive precursor and cleavage at the S1/S2 site leads to the formation of active S protein which is a prerequisite for fusion initiation [26]. Though the exact mechanism of SARS-CoV-2 virus entry and fusion to host cell is still intriguing, numerous studies propose that the spike protein cleavage and entry could occur via different mechanisms: (1) during the spike protein trafficking in the ER-golgi pathway, it could be cleaved by furin like proteases (2) endocytic uptake and cleavage processing inside the late endosomes by cathepsin L proteases, and (3) at the cell surface, where the spike protein can be cleaved and activated at the plasma membrane via serine protease, TMPRSS2 [27, 28]. Hence, the highly conserved cleavage site sequence plays an important role in SARS-CoV-2 entry, infectivity, and transmission. The SARS-CoV-2 evolutionary pathway suggests that the presence of D614G and P681R/H mutation might have a role in virus entry, and fusion. To understand the role of these salient mutations, we utilized the HIV-1/spike pseudovirus system to generate Wuhan-1, D614G, Alpha, Delta, Omicron, P681H single mutant and D614G + P681R double mutant pseudoviruses. Additionally, we have generated several other mutants consisting of D614G and several key mutations such as L452R, E484Q, G142D mutations. Here, we showed that the double mutant D614G + P681R results in enhanced infectivity, fusion, syncytial formation and marked increase in the virus entry by utilizing both TMPRSS2 dependent and independent spike activation, as similar to Delta virus, and consequently, indicates the impact of these two mutations in enhancing the infectivity. In contrast, the Omicron pseudovirus and live virus consisting of D614G and P681H mutations showed TMPRSS2 independent virus entry, low cell-to-cell fusion and a reduced infectivity titer as compared to the Delta variant. The presence of other key mutations (L452 or E484Q) in the D614G backbone also found to be enhanced the fusion and infectivity in synthetic D614G + L452R, D614G + L452R + E484Q mutants as compared to the Wuhan-1, thus suggesting that these are the beneficial mutations in the SARS-CoV-2 spike domain. Our findings further revealed that the single P681H mutant phenotypically behaves alike the Omicron variant. Similar findings were also observed with the Delta and Omicron live viruses. Taken together, our data suggests that the proline 681 mutation along with the D614G are one of the major determinants of viral entry and tropism to different host compartments.

## Results

### Variant of concern viruses and synthetic mutants spike expression and cleavability

**Fig. 1:**
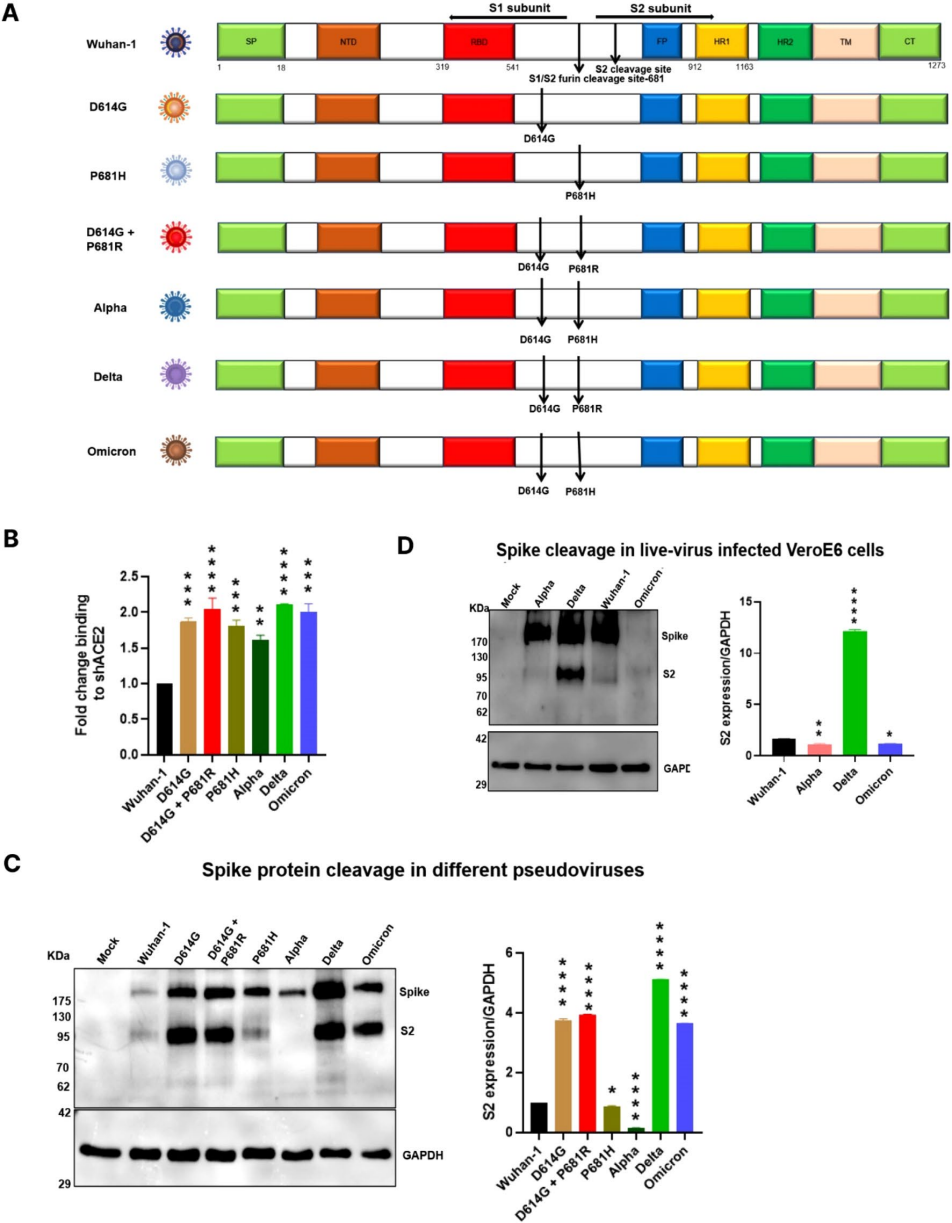
Expression and cleavage of SARS-CoV-2 variants and synthetic mutants. **A** Schematic representation of Wuhan-1, Alpha, Delta, and Omicron spike proteins with D614G and P681R/H mutations and synthetic mutants, the signal peptide (green box), S1-N terminal domain NTD (brown box), receptor binding domain (red box), fusion peptide (blue box), heptad repeat 1 (yellow box), heptad repeat 2 (dark green box), transmembrane domain TM (cream box), cytoplasmic tail CT (green box), S1/S2 and S2′ cleavage site, presence of D614G, P681H/R mutation. **B** Surface expressed spike protein binding to soluble-hACE2 was analyzed by expressing the spike of variants and mutants in HEK293T cells and 36 h post transfection incubated with soluble-hACE2 and analyzed by flow cytometry. Statistical significance was determined using *t* test keeping Wuhan-1 as the control. The experiment was repeated twice and the error bars show mean values with SEM. **C** Detection of spike protein cleavage in different pseudoviruses at 24 h post transfection. Briefly, the pseudoviruses were produced in 293 T cells pseudotyped with various S glycoproteins, 24 h post transfection cell lysates were prepared for the western blot analysis and probed with anti-spike (Wuhan-1) mouse polyclonal sera and anti-GAPDH antibodies. Statistical significance was determined using one-way ANOVA keeping Wuhan-1 as the control. The experiment was repeated three times and the error bars show mean values with SEM. D. SARS-CoV-2 live viruses were infected at 0.1 MOI in VeroE6 cells and at 24 h post infection cell extracts were collected by lysing with RIPA lysis buffer, and spike protein was detected by western blot analysis and probed with anti-spike (Wuhan-1) mouse polyclonal sera and anti-GAPDH antibodies. GAPDH was used as a loading control for both pseudovirus and live virus analysis. The experiment was repeated twice. Statistical significance was determined using one-way ANOVA keeping Wuhan-1 as the control (*P* < 0.05), where (*P* < 0.05), *P < 0.05, **P < 0.01, ***P < 0.001, ****P < 0.0001 were considered significant and *P* > 0.05 was considered nonsignificant (ns)

Site-directed mutagenesis was performed on the full-length wild-type Wuhan-1 spike protein to produce D614G, P681H single and D614G + P681R double mutants to generate pseudoviruses. Alpha variant spike was generated in-house, consisting of all mutations in the spike protein except the Y144 deletion; the wild-type Wuhan-1, Delta and Omicron spikes were synthesized, and the pseudoviruses were generated as described in the materials and methods section (Fig. 1A). The spike proteins of variants and mutants were well expressed in BHK-21-transfected cells (Fig. S1B). We assessed the expression of the spike protein of the variants and mutants on the cell surface w.r.t Wuhan-1 spike protein expression via flow cytometry. Compared to the Wuhan-1 spike protein, the expression of variants or mutants’ spike protein was found to be significantly increased (Fig. S2A). We then analyzed the binding of surface-expressed spike to soluble hACE2 by flow cytometry. The Delta, Omicron and D614G + P681R spikes showed an ~ twofold increase in binding to soluble hACE2 (Fig. 1B). Earlier, it was shown that the D614G mutation significantly alters the spike conformation, thus modulating the interaction with the hACE2 receptor, which in turn affects virus entry and infectivity [29]. We further observed in the single P681H mutant, there was also an increase in binding to hACE2, which suggests that this mutation could also alter the spike conformation. The other key mutations present in the Delta or Omicron variants might influence the spike conformation. Peacock et al. recently showed that the introduction of Q498R in the Alpha variant, which is also present in Omicron, increases binding to soluble hACE2 [30].

Next, we analyzed the cleavage of spike protein incorporated in pseudovirions at 24 h post-transfection. As shown in Fig. 1C, although in all variants the spike protein was cleaved at 24 h post transfection, w.r.t to Omicron, the S2 band was more distinct in Delta spike. The pseudoviruses were prepared in HEK293T cells, which contain inherent furin-like proteases. The similar observation, we have also found in the cleavage of spike proteins of Delta live viruses, 24 h post infection in Vero E6 cells (Fig. 1D), where more distinct and clearer S2 band was shown as compared to other live viruses. We have also conducted similar experiment in A549 cells, collected the samples at 48hpi and western blot analysis showed higher S2 expression in Delta live viruses as compared to Omicron viruses (Fig. S2B). These data suggest Omicron spike protein might have slow cleavage processing in the presence of furin as compared to Delta spike protein. A similar phenomenon was shown by Ueo et al., where Mumps virus fusion was inefficiently cleaved when expressed in HEK293T cells [31]. However, further studies need to be performed to support this phenomenon.

### The presence of D614G and P681R mutations significantly enhanced virus infectivity and cell-to-cell fusion

**Fig. 2:**
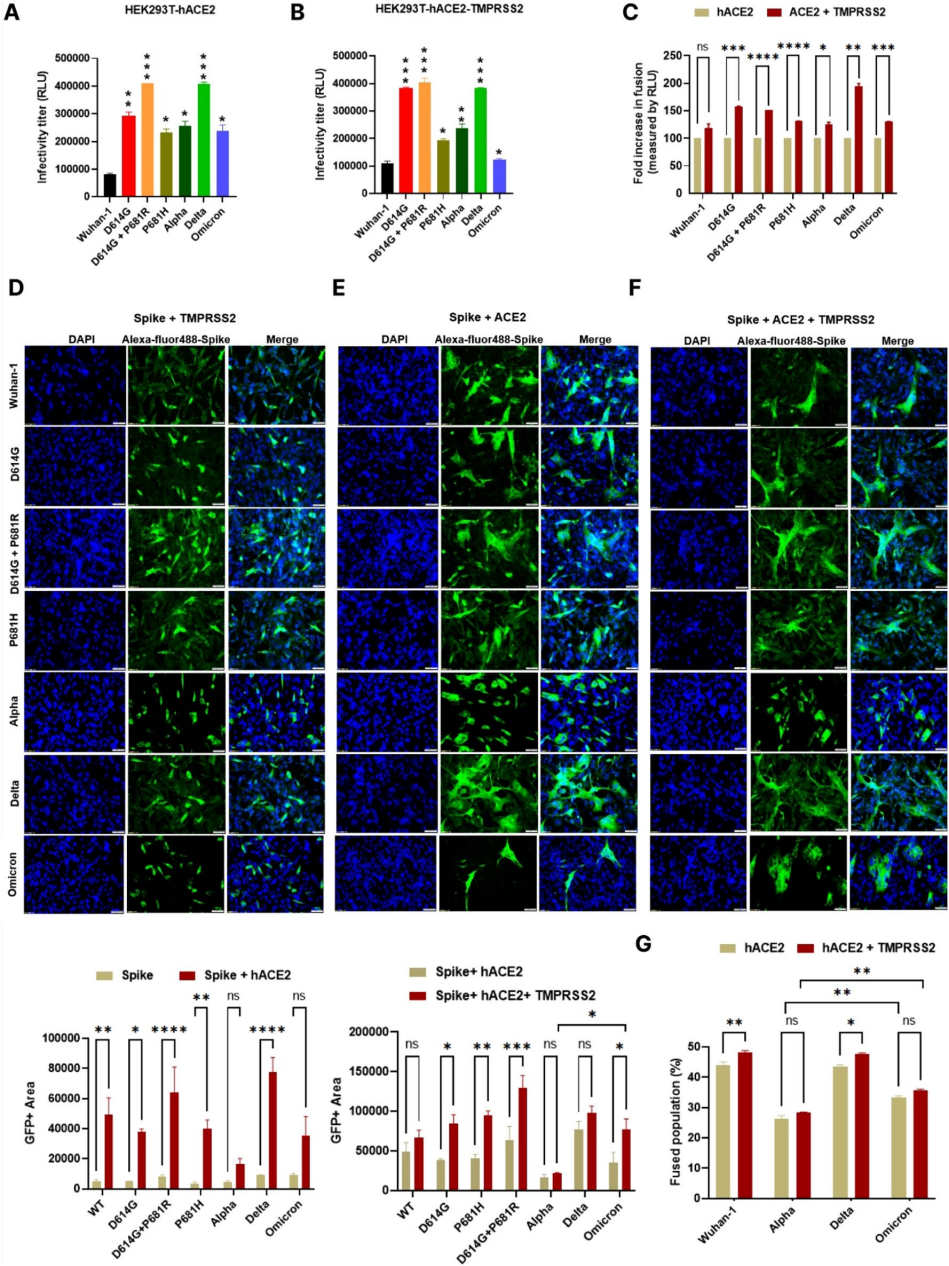
Infectivity titer and fusion of pseudovirus variants and mutants. **A** Pseudoviruses are produced in HEK293T cells, and infectivity was measured in the HEK293T cells overexpressing hACE2 (NR52511, BEI resources, USA). Statistical significance was determined using *t* test keeping Wuhan-1 as the control. The experiment was repeated three times and the error bars show mean values with SEM. **B** HEK293T cells overexpressing hACE2 and TMPRSS2 (HEK293T cells transfected TMPRSS2 plasmid and harvested 24 h post transfection and used in the assay) were infected with pseudoviruses, and the infectivity titer was measured 48 h post infection as relative luciferase units (RLU). Statistical significance was determined using *t* test keeping Wuhan-1 as the control. The experiment was repeated three times and the error bars show mean values with SEM. **C** Quantitative fusion assay in the presence of hACE2 or hACE2 + TMPRSS2 as measured by RLU. The data shown are the averages of three experiments in duplicates. Statistical significance was determined using two-way ANOVA, with multiple comparisons using hACE2 expression as control. The error bars show mean values with SEM. **D** Spike and TMPRSS2 plasmids were co-transfected into BHK-21 cells and probed with anti-Spike mouse polyclonal sera (1:200) and Alexa Fluor 488-labeled anti-mouse antibody (green) (1:1000) 36 h post transfection. E. Spike and hACE2 plasmids were co-transfected into BHK-21 cells, and fusion formation was shown by immunofluorescence. The data shown are the averages of three experiments in duplicates. Statistical significance was determined using two-way ANOVA, with multiple comparisons using spike expression as control. The error bars show mean values with SEM. F. Spike, hACE2 and TMPRSS2 plasmids were co-transfected into BHK-21 cells and fusion and syncytia formation was measured 24 h post transfection. The images were taken with an Olympus fluorescence microscope and quantification was done by comparing three randomly selected GFP + areas. The experiments were repeated three times. The nuclei were stained with 4′,6-diamidino-2-phenylindole (DAPI, blue); scale bar: 50 μm and magnification × 20. The data shown are the averages of three experiments in duplicates. Statistical significance was determined using two-way ANOVA, with multiple comparisons using hACE2 expression as control. The error bars show mean values with SEM. G. FACS based dual dye assay to measure fusion in the presence of hACE2 and hACE2 + TMPRSS2. Spike expressing cells were stained with orange dye, and hACE2 and hACE2 + TMPRSS2 cells with green dye. Orange and green dyed cells were incubated in equal ratio for 8 h, fixed and then acquired on BD FACS Canto II and analyzed on FlowJo. Statistical significance was determined using *t* test. The experiment was repeated three times and the error bars show mean values with SEM. Where (*P* < 0.05), *P < 0.05, **P < 0.01, ***P < 0.001, ****P < 0.0001 were considered significant and *P* > 0.05 was considered nonsignificant (ns)

Next, we examined the synthetic P681H, D614G + P681R and natural D614G, Alpha, Delta, and Omicron pseudovirus variants of their infectivity by measuring relative luciferase units in HEK293T cells overexpressing hACE2 and both hACE2 + TMPRSS2. As shown in Fig. 2A, the highest infectivity titers were found in D614G + P681R and Delta, followed by D614G > Alpha > Omicron > P681H in the HEK293T-hACE2 cell line. Additionally, Delta and D614G + P681R showed high infectivity in the HEK293T-hACE2-TMPRSS2-overexpressing cell line than in the Wuhan-1 pseudovirus and Omicron (Fig. 2B). Omicron pseudoviruses showed an ~ 2.18- to 1.52-fold rise in infectivity titer in hACE2 and hACE2 + TMPRSS2, respectively, w.r.t wild type pseudoviruses (Fig. 2A, B). These data suggest that as compared to the Delta variant, Omicron, and P681H mutant less efficiently use the TMPRSS2 dependent virus entry. This is corroborated by the study of Meng et al. who showed that the usage of the TMPRSS2 proteases was lower in the Omicron variant [32]. We further investigated fusion and syncytial formation by co-expressing spike variants with either hACE2 or TMPRSS2 or spike + hACE2 + TMPRSS2 in the BHK-21 cell line. Like earlier results, the highest fusion and syncytial formation was found in the Delta and D614G + P681R mutants (Fig. 2C) and when the spikes were co-expressed with TMPRSS2 alone, there was no fusion or syncytial formation (Fig. 2D). When the spike variants were co-expressed together with hACE2 and TMPRSS2, fusion and syncytial formation were markedly enhanced, and the highest values were observed in the Delta and D614G + P681R mutants (Fig. 2E, F). Additionally, a flow cytometry based dual dye assay was performed to calculate frequency of cell fusion, where spike expressing cells were stained with orange dye and hACE2/ hACE2 + TMPRSS2 expressing cells were stained with green dye (Fig. 2G). Both the cell types were incubated for 8 h in equal ratio and then cells were fixed and acquired. Gating strategy is shown in Fig. S5. Both Wuhan-1 and Delta viruses showed high cell fusion frequency as compared to Alpha and Omicron in the presence of TMPRSS2. We further assessed the enhancement of infectivity and fusion of Wuhan-1, Alpha, Delta, and Omicron pseudoviruses at different time course (0, 6, 12, 24 and 48 h) post infection. As shown in Fig. S3A, the highest infectivity titer was found in Delta pseudo viruses at 48 hpi in both hACE2 and hACE2 and TMPRSS2 overexpressed cells, followed by Alpha and Omicron pseudoviruses. Similar results were shown in cell-to cell fusion process, with highest fusion was seen in Delta pseudoviruses, at 24 hpi (Fig. S3B-C.). Interestingly, Alpha pseudoviruses showed low cell-to-cell fusion as compared to Wuhan-1 and Omicron at 24 hpi. These, data together might further explain the ability of Delta variant to exhibit high pathogenesis amongst the VoCs. The Delta virus could grow at high titers in the presence of ACE2 and in the lower respiratory lung cells where TMPRSS2 proteases are well-expressed, thus could be cleaved by various host cells compartments and might spread by both apical and cell-to-cell fusion. In contrast, Omicron might not efficiently be cleaved by TMPRSS2 proteases, thus shown favorable growth in few cellular compartments. Next we sought to assess, whether the presence of other key mutations in the spike protein [16, 33] might have beneficial, neutral, or compensatory effect in the fusion process and infectivity. We further generated several double and multiple mutants in the D614G backbone, such as D614G + L452R (designated as 3 M), D614G + E484Q (designated as 4 M) triple mutant D614G + E484Q + L452R (designated as 5 M), quadruple mutant D614G + E484Q + L452R + P681R (designated as 6 M), and penta-mutant consisting of D614G + E484Q + L452R + P681R + G142D mutation (designated as 7 M) (Fig. S4A, B) We evaluated the infectivity titer of these mutants in the presence of hACE2 and hACE2 + TMPRSS2 and compared with Wuhan-1 and D614G (Fig. S4C). Amongst the double and multiple mutants, the highest infectivity titer was shown in 5 M mutant (D614G + E484Q + L452R) followed by 3 M (D614G + L452R) mutant in both hACE2 and hACE2 + TMPRSS2 expressed cells. Notably, addition of P681R and G142D mutation did not enhanced the infectivity titer. All the mutants showed high fusion in 293 T-hACE2-TMPRSS2 cells as compared to 293 T-hACE2 cells, highest fusion shown in 3 M and 5 M mutants (Fig. S4D).

### P681R/H mutation modulates exogenous trypsin-mediated fusion and cleavage

**Fig. 3:**
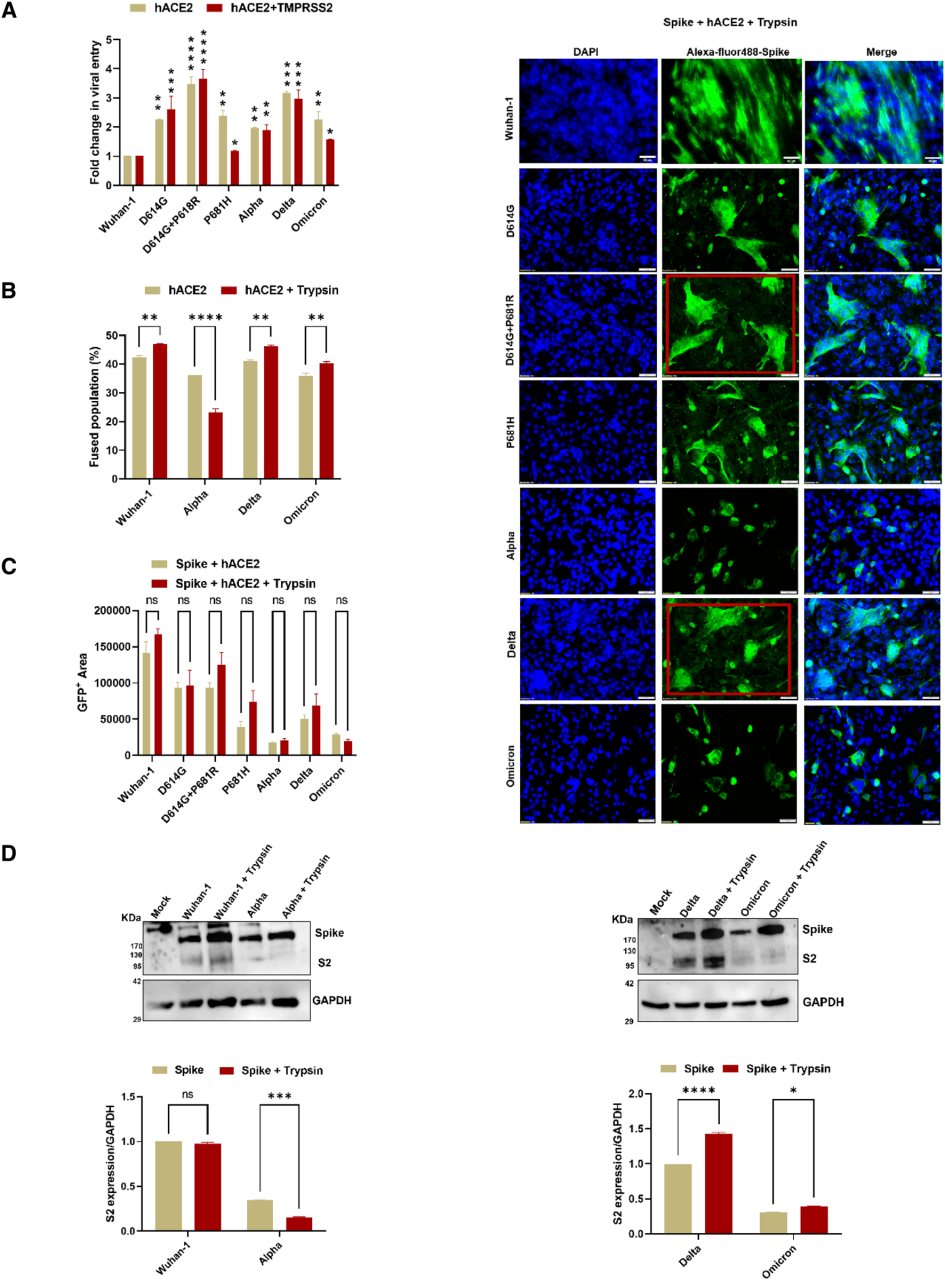
VoC and mutants pseudoviruses cell tropism and effect of trypsin in fusion and cleavage. **A** Pseudovirus entry was measured in HEK293T cells transfected with either hACE2 or hACE2 + TMPRSS2. Thirty-six h post transfection, HEK293T cells expressing hACE2 or hACE2 + TMPRSS2 were infected with variants and synthetic pseudoviruses, and relative luciferase titers were measured 48 h post transfection. The experiments were repeated two times in triplicates. Statistical significance was determined using two-way ANOVA, with multiple comparisons using Wuhan-1 as control. The error bars show mean values with SEM. **B** FACS based dual dye assay to measure fusion in the presence of hACE2 and hACE2 + Trypsin. Spike expressing cells were stained with orange dye, and hACE2 and hACE2 + Trypsin (2 mg/ml) cells with green dye. Orange and green dyed cells were incubated in equal ratio for 8 h, fixed and then acquired on BD FACS Canto II and analyzed on FlowJo. The experiment was repeated three times. Statistical significance was determined using two-way ANOVA, with multiple comparisons using hACE2 expression as control. The error bars show mean values with SEM. **C** Fusion and syncytial formation as measured by immunofluorescence assay in BHK-21 cells co-transfected with spike and hACE2 plasmids in the presence of trypsin. The experiment was repeated two times. Statistical significance was determined using two-way ANOVA, with multiple comparisons using spike + hACE2 expression as control. The error bars show mean values with SEM. D. Cleavage of Wuhan-1, Alpha, Delta and Omicron variant spike proteins in the presence and absence of trypsin as analyzed by Western blot after normalizing with GAPDH signal. The experiment was repeated three times. The error bars show mean values with SEM. Statistical significance was determined by using *t* test using spike expression without trypsin as control. Where *P* < 0.05, *P < 0.05, **P < 0.01, ***P < 0.001, and ****P < 0.0001 were considered significant and *P* > 0.05 was considered nonsignificant (ns)

As compared to the Wuhan-1 pseudoviruses, Delta variant and synthetic D614G + P681R mutant showed > threefold increase in virus entry in HEK293T-hACE2 and HEK293T-hACE2 + TMPRSS2 cells (Fig. 3A). In the Omicron, Alpha and P681H mutants, the virus entry was markedly higher in the hACE2 cell line than in hACE2 + TMPRSS2. The presence of proline in the SARS-CoV-2 S1/S2 cleavage site “SPRRAR-SVAS” was found to be conserved not only in MERS-CoV and HCoV-NL63 but also in the influenza cleavage site sequence (Fig. S6A). Ord et al. and group hypothesized that in the Wuhan-1 virus spike protein, the presence of serine followed by proline in the cleavage site allows proline-directed phosphorylation, which could modulate furin-directed cleavage in the ER-Golgi pathway and fusion capacity [34]. We further sought to assess the effect of proline on arginine or histidine mutations in spike variants in fusion and syncytia formation in the presence of exogenous trypsin. In the FACS based dual-color dye assay, increase fuse population of spike expressed cells were shown in Wuhan-1, Delta as compared to Omicron spikes in the presence of exogenous trypsin (Fig. 3B). Similar observation was also found in the fusion assay; in the presence of exogenous trypsin, there was a significant increase in fusion and syncytial formation in the D614G + P681R and Delta variants; as compared to Alpha, Omicron or P681H spike variants, thus suggesting that the P681R mutation is more favorable for cell-to-cell fusion in the presence of exogenous trypsin than P681H mutation (Fig. 3C). The presence of an additional basic residue R in the Delta variant “SRRRAR-SVAS” might allow more fusion in the presence of trypsin-like proteases. As shown in Fig. 3D, the addition of trypsin enhanced the cleavability of the Delta variant pseudoviruses as compared to the Omicron variant. Next, we performed the live virus infection in the VeroE6 cells in the presence of trypsin and measured the virus fusion and replication by qRT-PCR and plaque assay (Fig. S6B, D). In the presence of trypsin, Delta live virus showed significant increase in the virus copy number and formed higher number of plaques in Vero E6 cells; whereas Omicron live virus showed mild increase in virus copy number in the presence of trypsin.

### SARS-CoV-2 Wuhan-1 and VoC entry and fusion in the presence of E64d inhibitor and NH_4_Cl

**Fig. 4:**
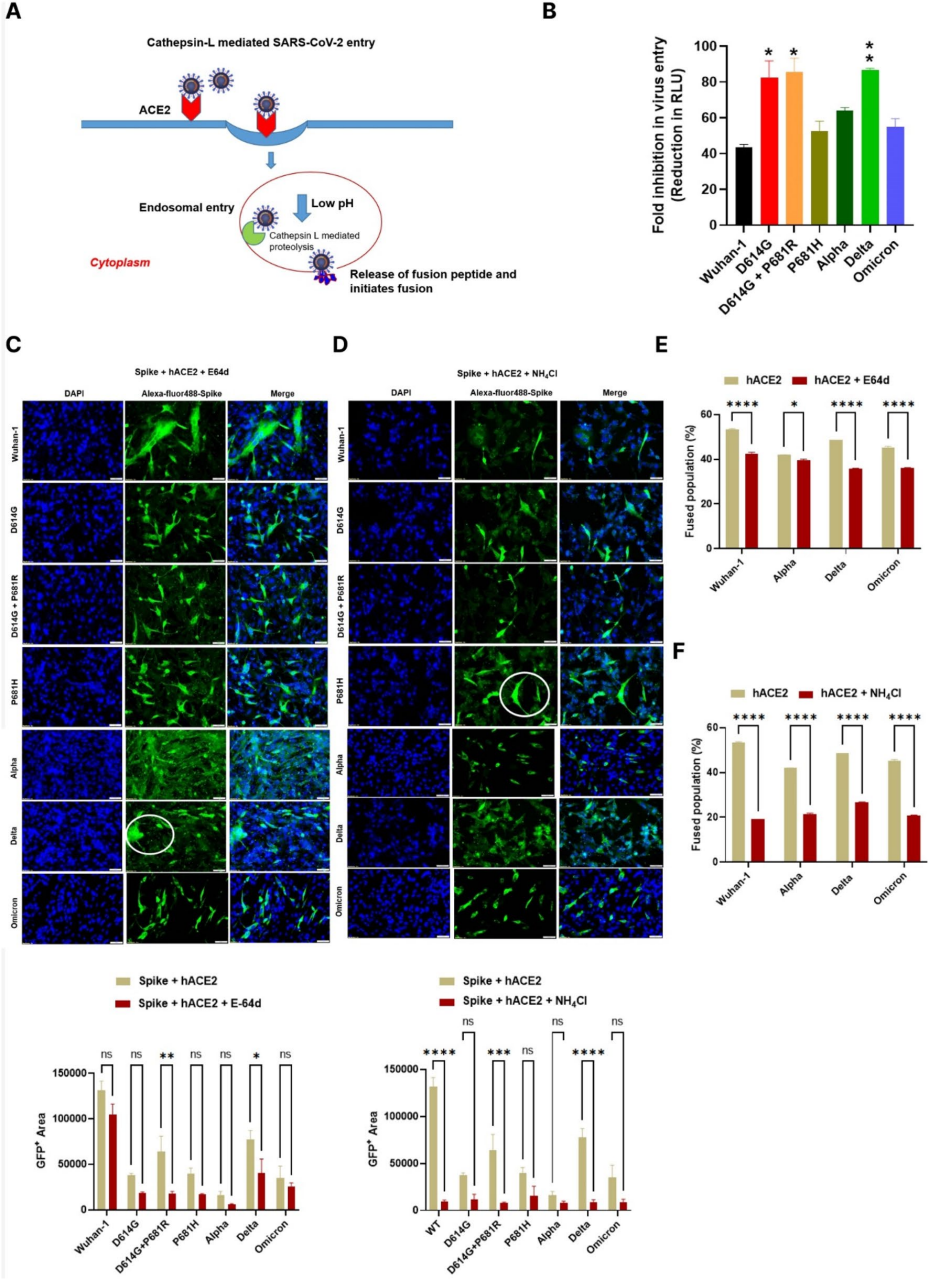
Analysis of variant entry and fusion in the presence of cysteine protease inhibitor. **A** Schematic representing hACE2-mediated endosomal entry and activation by cysteine protease in endosomes. **B** Pseudovirus entry in the absence or presence of the cysteine protease inhibitor E64d. The data represent mean of two experiments repeated in triplicate. Statistical significance was determined using two-way ANOVA, with multiple comparisons using hACE2 expression as control. The error bars show mean values with SEM. **C** Fusion of variants of spike proteins co-expressed with hACE2 in the presence of E64d in BHK-21 cells. The experiment was repeated three times. Statistical significance was determined using two-way ANOVA, with multiple comparisons using hACE2 expression without E-64d as control. The error bars show mean values with SEM. **D** Fusion of variants spike proteins co-expressed with ACE2 in the presence of NH_4_Cl in BHK-21 cells. The spike protein was probed with primary anti-Spike mouse polyclonal sera (1:200) (Wuhan-1 spike protein) and Alexa Fluor 488-labeled anti-mouse secondary antibody (green) (1:1000) at 24 h post transfection. The images were taken with an Olympus fluorescence microscope. The nuclei were stained with 4′,6-diamidino-2-phenylindole (DAPI, blue); scale bar: 50 μm and magnification × 20. The experiment was repeated three times. Statistical significance was determined using two-way ANOVA, with multiple comparisons using hACE2 expression without NH_4_Cl as control. The error bars show mean values with SEM. **E**, **F** FACS based dual dye assay to measure fusion in the presence of hACE2 and hACE2 + E-64d/NH4Cl. Spike expressing cells were stained with orange dye, and hACE2 and hACE2 + E-64d/NH4Cl cells with green dye. Orange and green dyed cells were incubated in equal ratio for 8 h, fixed and then acquired on BD FACS Canto II and analyzed on FlowJo. Statistical significance was determined using two-way ANOVA with multiple comparisons using ACE2 expression without inhibitor as control. Where *P* < 0.05, *P < 0.05, **P < 0.01, ***P < 0.001, and ****P < 0.0001 were considered significant and *P* > 0.05 was considered nonsignificant (ns)

**Fig. 5:**
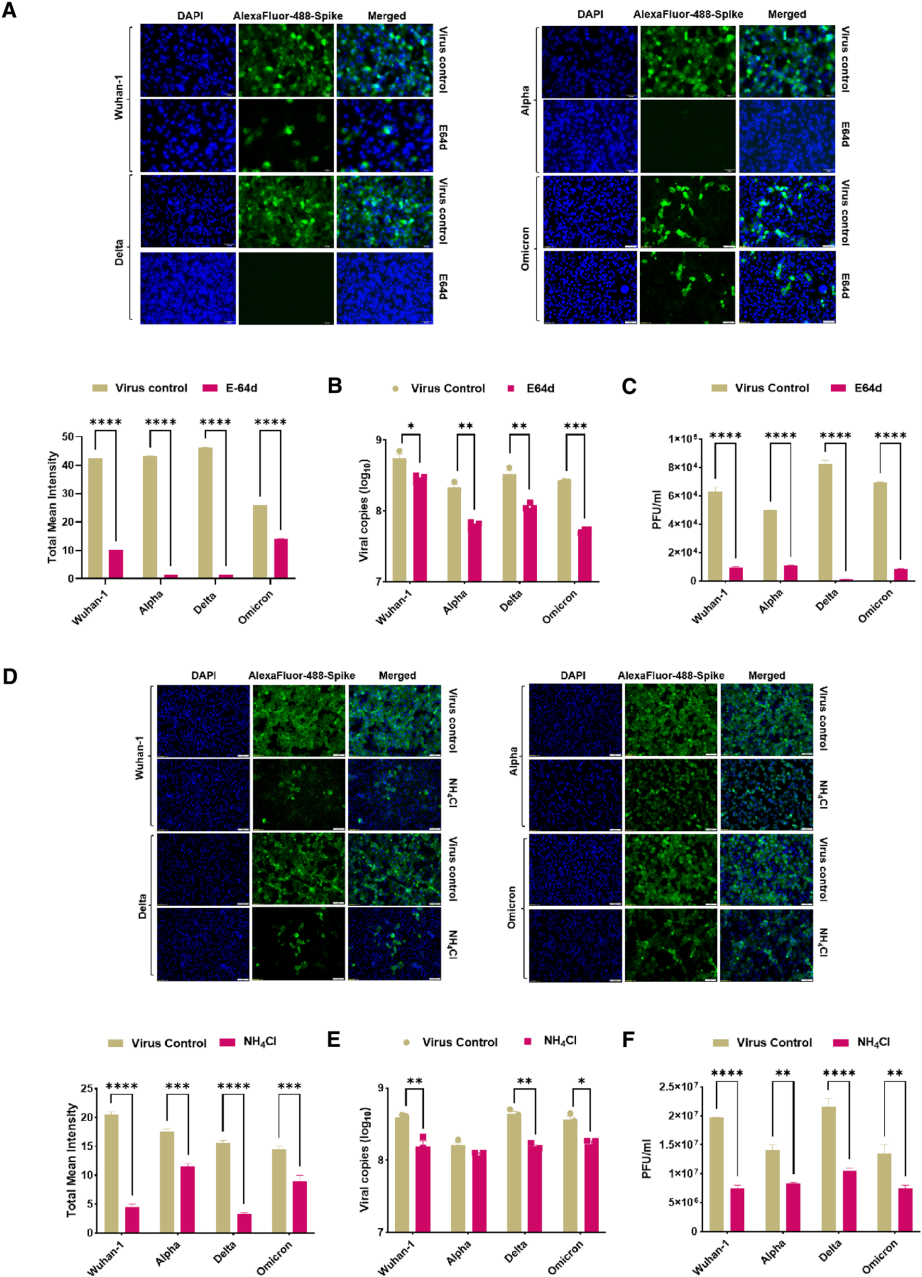
Effect of E64d and NH_4_Cl in Wuhan-1 and VoC live viruses’ infection. **A** Immunofluorescence assay for SARS-CoV-2 variants’ infection in VeroE6 cell line in the absence or presence of the inhibitor E64d. Briefly, live viruses were infected in Vero E6 cells with 0.1 MOI And 24 h post infection, cells were fixed with 4% paraformaldehyde and the SARS-CoV-2 spike protein was probed with primary anti-Spike mouse polyclonal sera (1:200) (Wuhan-1 spike protein) and Alexa Fluor 488-labeled anti-mouse secondary antibody (green) (1:1000). The images were taken with an Olympus fluorescence microscope. The nuclei were stained with 4′,6-diamidino-2-phenylindole (DAPI, blue); scale bar: 50 μm and magnification 20 × . The experiment was repeated three times. Statistical significance was determined using two-way ANOVA, with multiple comparisons using virus control as control. The error bars show mean values with SEM. **B**, **C**. VeroE6 cells were seeded and infected as mentioned above in the presence of E-64d. Cell lysates were harvested with trizol post 48 h of infection and relative copy number of SARS-CoV-2 N gene was estimated by quantitative RT-PCR keeping GAPDH or β-actin genes as an endogenous control for normalization. Cell supernatant was serially diluted and VeroE6 cells were infected to determine plaque forming units (Pfu/ml). The experiment was repeated three times. Statistical significance was determined using two-way ANOVA, with multiple comparisons using virus control as control. The error bars show mean values with SEM. **D** Immunofluorescence assay for SARS-CoV-2 variants’ infection in VeroE6 cell line in the absence or presence of the NH4Cl. Live viruses were infected in Vero E6 cells with 0.1 MOI and 24 h post infection, cells were fixed, stained and imaged as mentioned above. The experiment was repeated three times. Statistical significance was determined using two-way ANOVA, with multiple comparisons using virus control as control. The error bars show mean values with SEM. **E**, **F** VeroE6 cells were seeded and infected as mentioned above in the presence of NH4Cl. Cell lysates were harvested with trizol post 48 h of infection and relative copy number of SARS-CoV-2 N gene was estimated by quantitative RT-PCR keeping GAPDH or β-actin genes as an endogenous control for normalization. Cell supernatant was serially diluted and VeroE6 cells were infected to determine plaque forming units (Pfu/ml). The experiment was repeated three times. Statistical significance was determined using two-way ANOVA, with multiple comparisons using virus control as control. The error bars show mean values with SEM. Where *P* < 0.05, *P < 0.05, **P < 0.01, ***P < 0.001, and ****P < 0.0001 were considered significant and *P* > 0.05 was considered nonsignificant (ns)

It has been previously reported that SARS-CoV-2 in the presence of the ACE2 receptor can be rapidly endocytosed and cleaved by cathepsin L proteases in the endosomal compartment [35] (Fig. 4A). We assessed the entry of pseudovirus variants when treated with the cysteine protease inhibitor E64d [36, 37]. As shown in Fig. 4B, virus entry was not significantly reduced in D614G, D614G + P681R and Delta variant. Whereas, the Omicron, Alpha and P681H spike mutants showed ~ 55%, 63% and 52% inhibition of virus entry in the presence of E64d. However, Delta and D614G + P681R variant, showed significant reduction in fusion in the presence of E64d (Fig. 4C). Next, we evaluated the fusion of variants’ spike protein when cells were treated with the lysosomotropic agent ammonium chloride (NH_4_Cl). Ammonium chloride helps in endosomal acidification, which in turn modulates cathepsin L-mediated proteolytic activity [37]. In the presence of 20 mM ammonium chloride, a reduction in fusion was observed in all the variants and in the Wuhan-1 pseudovirus, highest in the Delta and D614G + P681R variant (Fig. 4D). Similar observation was shown in FACS based dual dye test, there was decrease in number of spikes expressed fuse cells shown in Delta and Wuhan-1 spike in the presence of E64d and ammonium chloride (Fig. 4E, F). Omicron spike also showed decrease in spike expressed fuse cells in the presence of ammonium chloride. Next, we tested the effect of E64d in the live viruses’ infection. As shown in Fig. 5, Delta live virus showed complete inhibition of fusion as compared to Omicron live viruses. We also assessed the live virus replication in the presence of E64d and NH_4_Cl. As shown in Fig. 5B and C Wuhan-1, Alpha, Delta, and Omicron live viruses showed substantial decrease in the virus copies number and plaque forming units in infected VeroE6 cell lines in the presence of E64d. In the presence of NH_4_Cl, there was also a decrease in cell-to-cell fusion and virus replication in the infected Vero E6 cells (Fig. 5D–F). The Delta virus showed marked inhibition of viral copy numbers and plaques as compare to Omicron and Alpha VoC.

### Effect of TMPRSS2 protease inhibitor on pseudovirus variant entry and fusion

**Fig. 6:**
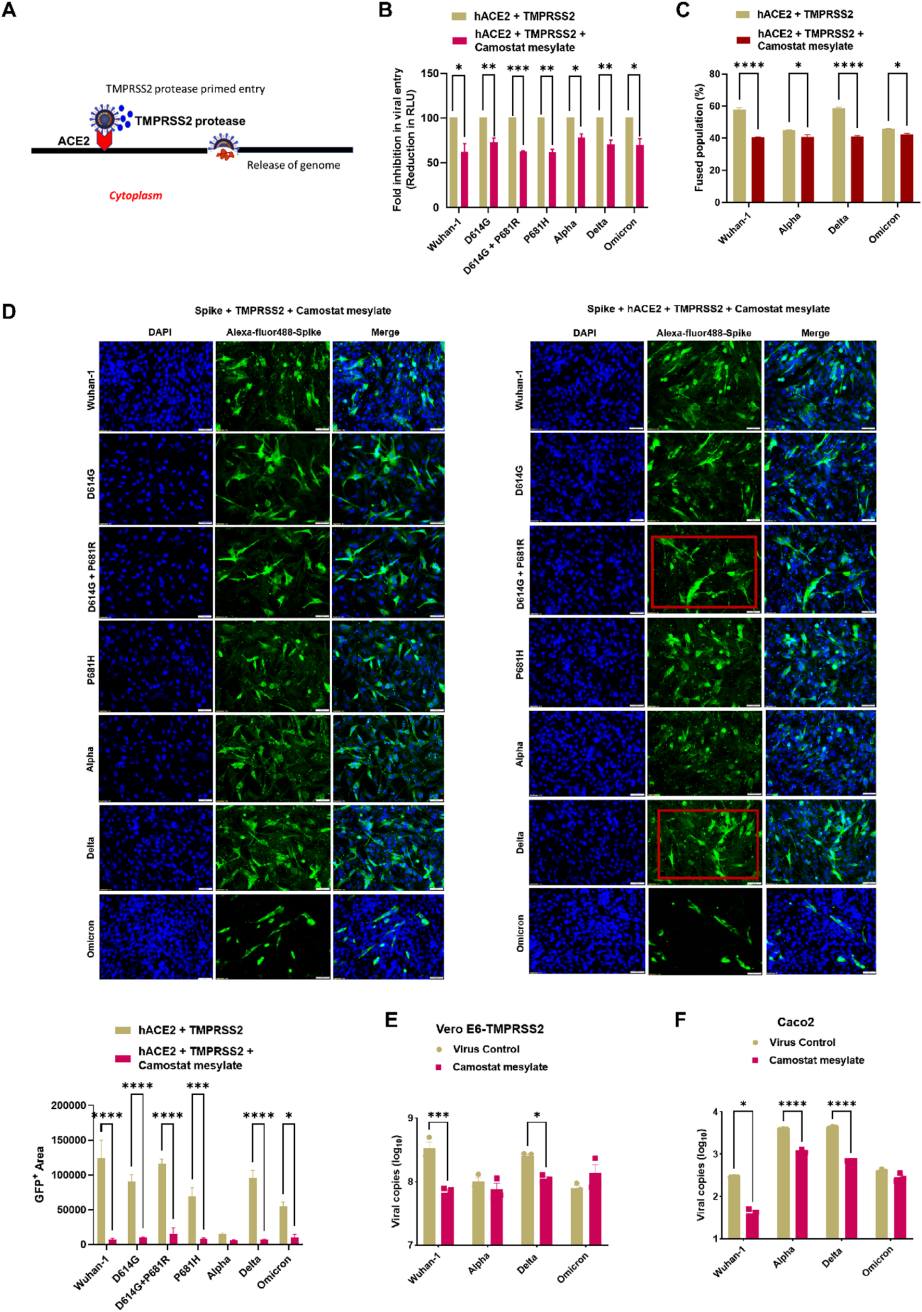
Analysis of variant entry and fusion in the presence of the serine protease TMPRSS2 inhibitor. **A** Graphical representation of TMPRSS2 mediated spike cleavage and cell entry. **B** Pseudovirus entry in the absence or presence of Camostat mesylate inhibitor when spike co-expressed along with hACE2 and TMPRSS2. The experiments were repeated three times. Statistical significance was determined using two-way ANOVA, with multiple comparisons using hACE2 + TMPRSS2 as control. The error bars show mean values with SEM. **B** Fusion of variants of spike proteins co-expressed with TMPRSS2 in the presence of Camostat mesylate in BHK-21 cells. **C** FACS based dual dye assay to measure fusion in the presence of hACE2 + TMPRSS2 + Camostat mesylate. Spike expressing cells were stained with orange dye, and hACE2 + TMPRSS2 + Camostat mesylate cells with green dye. Orange and green dyed cells were incubated in equal ratio for 8 h, fixed and then acquired on BD FACS Canto II and analyzed on FlowJo. The experiments were repeated three times. Statistical significance was determined using *t* test using hACE2 + TMPRSS2 as control. The error bars show mean values with SEM. D. Fusion of variants spike proteins co-expressed with TMPRSS2 and or hACE2 + TMPRSS2 in the presence of Camostat mesylate in BHK-21 cells. The spike protein was probed with primary anti-Spike mouse polyclonal sera (1:200) (Wuhan-1 spike protein) and Alexa Fluor 488-labeled anti-mouse secondary antibody (green) (1:1000) at 24 h post transfection. The images were taken with an Olympus fluorescence microscope. The experiments were repeated three times. The nuclei were stained with 4′,6-diamidino-2-phenylindole (DAPI, blue); scale bar: 50 μm and magnification × 20. The experiments were repeated three times. Statistical significance was determined using two-way ANOVA, with multiple comparisons using hACE2 + TMPRSS2 as control. The error bars show mean values with SEM. **E**, **F** VeroE6 overexpressing TMPRSS2 and Caco2 cells were seeded and infected as mentioned earlier in the presence of Camostat mesylate. Cell lysates were harvested with trizol post 48 h of infection and relative copy number of SARS-CoV-2 N gene was estimated by quantitative RT-PCR keeping GAPDH or β-actin genes as an endogenous control for normalization. The experiments were repeated two times. Statistical significance was determined using two-way ANOVA, with multiple comparisons using hACE2 + TMPRSS2 as control. The error bars show mean values with SEM. Where *P* < 0.05, *P < 0.05, **P < 0.01, ***P < 0.001, and ****P < 0.0001 were considered significant and *P* > 0.05 was considered nonsignificant (ns)

We further evaluated the effect of Camostat mesylate, which is a TMPRSS2 serine protease inhibitor (Fig. 6A), on pseudovirus variant entry and fusion. The D614G, Delta and D614G + P681R pseudovirus variants showed more than a > 60% reduction in virus entry into cells co-expressing ACE2 and TMPRSS2 in the presence of 100 µM Camostat mesylate (Fig. 6B). Omicron, P681H and Alpha variant pseudoviruses showed less than < 50% reduction in virus entry in the presence of Camostat mesylate. In the FACS based dual dye assay, in the presence of Camostat mesylate, the Wuhan-1, Delta spikes showed marked decrease in fused cells population as compared to Omicron spike (Fig. 6C). In BHK-21 cells, the spike proteins of different variants were co-expressed either in the presence of TMPRSS2 alone or with ACE2 and TMPRSS2 together. Upon the addition of Camostat mesylate, surprisingly fusion or syncytial formation was found to be diminished in all variants (Figs. 6D, S1D). We further investigated the effect of Camostat mesylate in live virus infection. We overexpressed TMPRSS2 in Vero E6 cells and infected the cells with Wuhan-1, Alpha, Delta, and Omicron live viruses and treated with 100 µM Camostat mesylate as discussed in the materials and methods. The Wuhan-1 and Delta viruses showed reduction in virus replication as measured by qRT-PCR (Fig. 6E). We also checked the virus replication in intestinal epithelial Caco2 cells, which expresses TMPRSS2 [38]. In the Caco2 cells, Wuhan-1, Alpha, and Delta viruses showed reduction in virus replication in the presence of Camostat mesylate (Fig. 6F); however, Camostat mesylate did not inhibit Omicron live viruses’ replication in both the cell lines in the presence of TMPRSS2.

### Omicron pseudovirus stability, S1 shedding and cross reactivity to recombinant soluble Delta RBD polyclonal sera

**Fig. 7:**
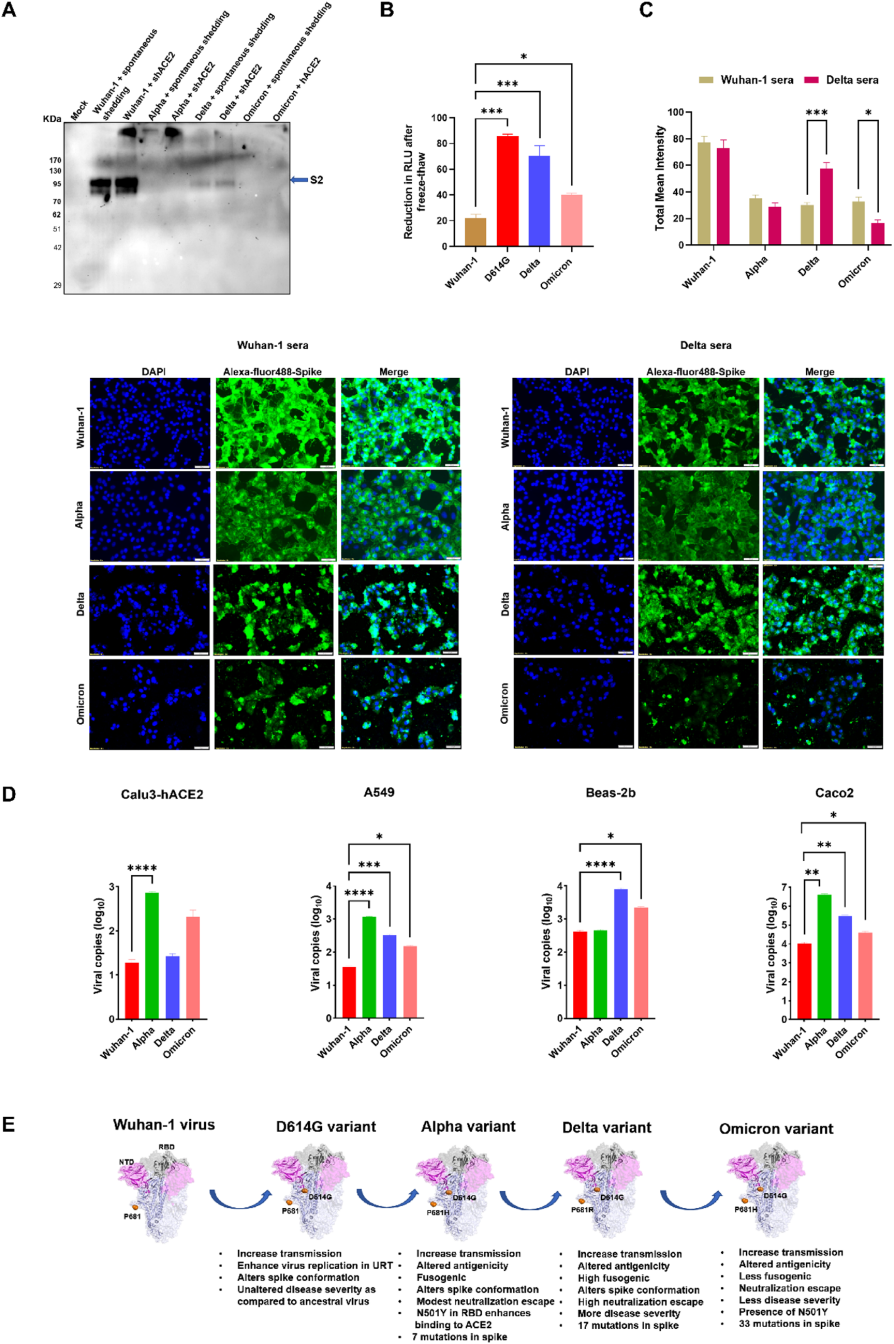
Stability, shedding, antigenicity and characteristics of variants with D614G and P681R/H mutations. **A** Assessment of soluble-hACE2 induced and spontaneous shedding of the S1 domain as measured by Western blot. **B** Measurement of infectivity titers of Wuhan-1, D614G, Delta and Omicron pseudoviruses in HEK293T-hACE2 cell lines after two freeze–thaw cycles. The experiments were repeated two times. Statistical significance was determined using one-way ANOVA, with multiple comparisons using Wuhan-1 as control. The error bars show mean values with SEM. **C** Cross reactivity of Omicron spikes to anti-Wuhan-1 RBD and anti-Delta RBD polyclonal mouse sera as measured by immunofluorescence in BHK-21 cells. Briefly, spike protein was transfected into the BHK-21 cell line, and 24 h post transfection, the cells were fixed and probed with anti-RBD (Wuhan-1) or anti-Delta RBD polyclonal mouse sera (1:200 dilution) and Alexa Fluor 488-labeled anti-mouse secondary antibody (green) (1:1000 dilution). The images were taken with an Olympus fluorescence microscope. The nuclei were stained with 4′,6-diamidino-2-phenylindole (DAPI, blue); scale bar: 50 μm and magnification × 20. The experiments were repeated two times. Statistical significance was determined using two-way ANOVA, with multiple comparisons using Wuhan-1 sera as control. The error bars show mean values with SEM. **D** Calu3-hACE2, A549, Beas-2b and Caco-2 cells were infected with Wuhan-1, Alpha, Delta, and Omicron viruses at 0.1 MOI. Cell lysates were harvested with trizol post 48 h of infection and relative copy number of SARS-CoV-2 N gene was estimated by quantitative RT-PCR keeping GAPDH or β-actin genes as an endogenous control for normalization. The experiments were repeated three times. Statistical significance was determined using one-way ANOVA, with multiple comparisons using Wuhan-1 as control, where (*P* < 0.05), *P < 0.05, **P < 0.01, ***P < 0.001, and ****P < 0.0001 were considered significant and *P* > 0.05 was considered nonsignificant (ns). The error bars show mean values with SEM. **E** Structural modeling of the spike protein was created by using PYMOL software. Pink represents the N-terminal domain and gray RBD domain, orange dots, D614G and P681H/R mutations. The evolution of virus and variant characteristics are schematically represented

Zhang et al. showed that the D614G mutation allowed a decrease in S1 shedding and thus the presence of a more functional whole spike virion, which enhances infectivity [39]. Hence, we sought to test the S1 shedding of Omicron pseudoviruses when kept at room temperature and in the presence of soluble hACE2. As shown in Western blot analysis (Fig. 7A), in the Wuhan-1 spike protein, S1 shedding was more pronounced, whereas no S2 band was seen in the Alpha and Omicron pseudovirus supernatant. A faint band of S2 was seen in the Delta pseudovirus supernatant. Next, we tested the stability of the Omicron spike pseudoviruses, when subjected to repeated freeze–thaw and then measured the infectivity titer. As compared to Delta and D614G variants, the Omicron pseudovirions infectivity titer was impaired when subjected to two freeze–thaw cycles (Fig. 7B). We further investigated the ability of the polyclonal sera raised in BALB/c mouse against recombinant soluble Wuhan-1 and Delta RBD protein to recognize the VoC spike proteins when expressed either in the BHK-21 cell line or live virus infected in VeroE6 cell line (Figs. 7C, S7A.). Live-virus based ELISA was also performed for the same where, after infection cells were fixed and stained with Wuhan-1 and Delta RBD sera as above and further stained with goat anti-mouse HRP secondary antibody (Fig. S7B). The Omicron live viruses were found to be slow growing at 24 hpi, and recognized by Wuhan-1 or Delta RBD mouse polyclonal sera. Future experiments are planned to assess the cross neutralization of immunized sera with different soluble RBD proteins against different VoC viruses, which could provide greater clarity on the effect of antigenicity and immunogenicity. Furthermore, we performed the live virus replication in different epithelial cell lines (respiratory epithelial cell lines Calu3-over expressing ACE2, A549, bronchial epithelial cells BEAS-2b and intestinal epithelial cells Caco-2) (Fig. 7D). In the respiratory epithelial cells (1) Calu3-over expressed with hACE2, highest virus titer was shown in Alpha > Omicron > Delta > Wuhan-1; (2) in A549 the highest virus titer was shown in Alpha > Delta > Omicron, where as in bronchial epithelial cells highest virus titer was shown in (3) Delta followed by Omicron. The intestinal epithelial cells Caco2 mostly favored the growth of Alpha followed by Delta > Omicron. These results strongly suggest the favorable and restricted growth of Omicron viruses mainly in the respiratory epithelium, whereas viruses like Alpha and Delta could efficiently replicate both in respiratory and intestinal epithelial cells.

Taken together, our results elucidate the interplay between the evolution of SARS-CoV-2 with the introduction of D614G, cleavage site proline mutation and additional key mutations, which are together impacting the virus entry, replication and thus might be modulating the virus transmission and pathogenesis (Fig. 7E).

## Discussion

With the emergence of novel SARS-CoV-2 and its variants of concern, new pandemic wave continues to impact enormous economic loss and crippling the health care system all over the world. The introduction of new mutations in the SARS-CoV-2 virus is mainly in the spike domain, which results in immune escape and a drastic reduction in neutralization efficacy to current vaccines [40–43]. Hence, it is utmost important to understand the role of key mutations in the virus entry and fusion mechanism of emerging variants. SARS-CoV-2 is an enveloped RNA virus, and studies from important other RNA viruses, such as influenza and HIV-1, have shown that RNA viruses selectively introduce mutations for virus transmission, infectivity and fitness [44] and the mutants with deleterious fitness mutations slowly disappear. Tracking the major SARS-CoV-2 variants of concern has shown the presence of D614G and cleavage site P681R/H mutations in the spike protein, which might be an indicator of infectivity and disease transmission (Fig. 1A).

The introduction of mutations in the spike region changes the conformation, as shown previously with D614G, Alpha and Delta variants, thus modulating the binding to the hACE2 receptor, which in turn determines virus entry and immune escape to vaccines [45–47]. Here, we have shown that variants including Omicron spike and synthetic spike mutants expressed on the surface bind to soluble-hACE2 at a higher fold than the Wuhan-1 spike protein (Fig. 1B), which corroborates other reported studies [48, 49]. The synthetic double mutant D614G + P681R showed a high infectivity titer in both ACE2- and ACE2-TMPRSS2-overexpressing cells, thus indicating the potential role of these two mutations in cleavage site activation by different cellular proteases. In addition, assessment of the fusion and syncytial formation of these spike variants and synthetic mutants further showed that the presence of the D614G and P681R mutations significantly enhanced the fusogenic property and syncytial formation (Fig. 2B–E). Interestingly, there is a marked reduction in the fusion capacity of Omicron pseudoviruses. The presence of the P681H mutation is highly conserved in the Omicron and its subsequent lineages BA.1, BA.2, BA.3, BA.4 and BA.5 (Fig. S1A), which might be attributing to the low fusogenic property of the Omicron spike protein (Fig. 3). However, the presence of other key mutations in the VoC spike domain might also facilitate the virus entry and infectivity. L452R and E484Q mutation are critical mutations in the spike protein found in the Delta variants (B.1.617.1, B.1.617.2) and presence of these mutations have shown to increase virus infectivity [50–52]. In our study, synthetic mutants D614G + L452R and D614G + L452R + E484Q have also shown higher infectivity and cell-to-cell fusion using pseudovirus assay.

The presence of a multibasic cleavage site sequence in viral envelopes allows efficient cleavage by furin-like proteases [26] and determines the virulence [53]. Further assessment of SARS-CoV-2 variant spike proteins has shown that the P681R mutation allows increased cell-to-cell fusion in the presence of exogenous trypsin, as shown in Delta and double D614G + P681R mutant spike and has less effect in Omicron spike, which has the P681H mutation and in the single P681H mutant (Fig. 3C). These data suggest that the presence of the P681H mutation in the Omicron and its lineages spike protein cleavage site might be downregulating the cleavability of the spike protein by different host cell proteases, thus restricting the localized replication of Omicron virus. The Delta live viruses also showed enhance replication in VeroE6 cells in the presence of exogenous trypsin as compared to Omicron live viruses (Fig. S6).

Peacock et al. and group have recently shown [30] that Omicron viruses use the TMPRSS2-independent entry pathway. In our study, we also found that even Omicron has preferentially entry into host cells by TMPRSS2 independent pathway, and Omicron pseudovirus entry could be reduced to ~ 55% in the presence of E64d, whereas in the presence of the TMPRSS2 inhibitor Camostat mesylate, there was only an ~ 34% reduction in virus entry (Figs. 4, 5, 6), where as in contrast Delta pseudoviruses are less efficiently effective against E64d. Previous studies on SARS-CoV have reported that the entry of viruses into the lungs is mainly mediated by TMPRSS2 proteases, which is highly expressed in the lower respiratory tract [54]. The presence of P681R in the Delta cleavage site adds another basic residue in the cleavage site, and the serine amino acid that is present near the cleavage site might be more accessible by serine proteases than the Omicron spike cleavage site, in which P-681 was replaced by histidine. Moreover, serine and other proteases require accessibility to the furin recognition motif and extended loop length for proper proteolytic cleavage [55]. Mutation of P681 to R might allow better accessibility of both intra- and extracellular proteases to act on the spike cleavage site. However, more studies are needed to temper this observation. Our assessment in the live viruses also showed the similar results. The Omicron live viruses showed significant decrease in virus replication and plaque forming units in the presence of E64d (Fig. 5A–C); where as in presence of TMPRSS2 protease inhibitor Camostat mesylate, there was no significant effect in Omicron live viruses’ replication (Fig. 6). These results further demonstrated the preferential entry of Omicron viruses via TMPRSS2 independent pathway [23].

In our findings, the variant spike proteins showed a marked rise in surface expression in HEK293T cells compared to the Wuhan-1 virus (Supplementary Fig. 2), further suggesting that the D614G and P681H/R mutations could efficiently support spike processing and transport through the ER-Golgi pathway to the surface. Spike density in the virus and stability of the spike protein are also major determinants of virus virulence and immune escape. D614G mutation have been shown to be more stable, and in the presence of hACE2, S1 shedding has been found to be reduced [56, 57]. Our study is in corroboration with earlier findings, and we found that even after repeated freeze–thaw cycles, the variants are more stable than the Wuhan-1 virus (Fig. 7). Additionally, the soluble-hACE2-induced shedding of the S1 domain was lower in variants than in the wild-type spike protein (Fig. 7A). The VoC live viruses also showed differential replication pattern in respiratory and intestinal epithelial cells (Fig. 7D), suggesting the presence of key mutations like D614G, P681R/H or other key mutations could significantly change virus phenotypes and host cell tropism.

In summary, we showed that the presence of D614G and P681R mutations is crucial for SARS-CoV-2 variant virulence and pathogenesis, as seen with the Delta variant. In the Omicron virus, although the D614G mutation is present, the P681H mutation might have resulted in slow cleavage and allowed ACE2-dependent and TMPRSS2 independent entry, thus restricting Omicron virus replication mainly to the upper respiratory tract. Although we have not studied the role of other mutations present in the Omicron spike protein, the single synthetic mutant P681H phenotypically behaves more similarly to Omicron. The presence of other key mutations also showed to modulate virus infectivity and fusogenicity. Although the study has the limitations that we could not clearly demonstrate, the higher transmissibility of Omicron viruses, however, the study showed that in the presence of over expressed hACE2 receptor (Calu3-hACE2), the Omicron live viruses could efficiently replicate to high titers. Our results further demonstrated high replication of Omicron and Delta live viruses in bronchial epithelia cells. Recently, Hui et al. have demonstrated higher growth of Omicron viruses in the bronchi than lung parenchyma [58]. The presence of higher number of virus titers in the upper respiratory tract might allow more virus shedding and thus might facilitate rapid spread and high transmission. Understanding the virus entry pathway will further allow us to appropriately select cell lines for in vitro screening of drugs and vaccine candidates and the development of novel drugs. Variants of concern, such as Delta and Omicron, demonstrated the evolution of both virulent and moderately virulent strains for better fitness for survival.

## Materials and methods

### Plasmids, viruses, and cell lines

The full-length spike of the SARS-CoV-2 isolate Wuhan-Hu-1 (GenBank: MN908947.3, Surface glycoprotein) was codon optimized, cloned into the pcDNA3.1 vector and synthesized by Thermo Fisher Scientific, USA. HIV-1 pNL4-3.Luc. R-E-plasmid NIH AIDS reagent, a kind gift from Dr. Kalpana Luthra, AIIMS, India. The Vero E6 and BHK-21 cell lines were kind gifts from Dr. Sudhanshu Vrati, RCB, India. HEK293T, HEK293T-hACE2, Vero E6, and BHK-21 cells were cultured in DMEM with 10% fetal bovine serum (FBS), 1% glutamine and 1% penicillin/streptomycin and routinely tested for mycoplasma-free status. The live viruses were obtained from BEI resources.

### Pseudovirus production, infectivity titer and virus entry assay

Pseudoviruses were prepared as described previously [59]. The pseudoviruses were titered in the HEK293T-hACE2 cell line. For the infection titer, HEK293T cells overexpressing hACE2 or both HEK293T-hACE2 and TMPRSS2 were seeded into 100 µl of pseudovirus supernatant, and 48 h post infection, the cells were lysed with britelite plus reporter substrate (Perkin Elmer). The luminescence was measured by a PerkinElmer luminometer. For the virus entry study, 40,000 cells/well were seeded into 96-well plates, and after 4 h, equal viral supernatant that gave ~ 75,000 relative luciferase units were chosen for infection in this assay.

### Pseudovirus entry inhibitor assay

Forty thousand HEK293T cells were seeded in a 96-well plate. After 24 h, cells were co-transfected with plasmids encoding CoV S glycoprotein and PLN4 backbone expressing luciferase. After 24 h of transfection, ACE2 and ACE2 + TMPRSS2 over expressing HE293T cells were overlaid on previous day transfected HEK293T cells. E64d (20 µM) and Camostat mesylate (100 µM) were also added to the respected wells along with the cells. After 16 h, cells were lysed with britelite substrate and luminescence was measured by PerkinElmer luminometer.

### Western blot and FACS-based surface expression

Soluble hACE2, Delta RBD, and soluble prefusion spike proteins were expressed in transiently transfected Expi293F cells using Expi293Fectamine (Invitrogen) following the manufacturer’s protocol. HEK293T cells/VeroE6 (0.5 million/well) were seeded in a 6-well plate. Next day, cells were transfected/infected with the pseudovirus plasmids/live viruses for all the variants. After 24 h of transfection, cells were given a PBS wash and lysed with RIPA lysis buffer. After sonication at an amplitude of 10 for 2 min, the cell lysates were centrifuged at 10,000 rpm for 10 min at 4 °C. To the supernatant, protein loading dye was added. The samples were then heated at 100 °C for 5 min and separated in a 10% SDS-PAGE gel and analyzed by western blot. Spike and GAPDH were probed with anti-spike mouse polyclonal sera (1:500) and anti-GAPDH mouse monoclonal antibody (1:1000, Invitrogen, AM4300) respectively and HRP-conjugated anti-mouse secondary antibody (1:2000, Jackson ImmunoResearch, PA, USA).

For the trypsin-based spike cleavage analysis, the spike-transfected cells were treated with TPCK-trypsin (2 mg/ml) for one hour in DMEM only after 24 h of transfection and later incubated with 10% FBS-containing DMEM for one hour. After this treatment, cell lysate preparation and western blotting were performed as described above. FACS-based cell surface expression assays were carried out as described previously [60]. Anti-spike sera were raised in mice by immunizing 20 µg of SARS-CoV-2 soluble stabilized spike-His tag (NR-52394, BEI Resources) protein or other proteins along with AddaVax by 0-day prime, 28-day boost, anti-spike mouse polyclonal sera were collected after 14 days boost and used in all assays (Western blot, Immunofluroscence, FACS).

### Soluble hACE2 binding assay

For the hACE2-spike binding assay, HEK293T cells were transfected with spike plasmids, and 24 h post transfection, cells were harvested with 5 mM EDTA, washed twice with 5% FBS in 1 × PBS, and then incubated with 4 μg/ml soluble hACE2 for 1 h on ice. After incubation, the cells were washed with FACS buffer and incubated with polyclonal goat anti-human ACE2 antibody (1:200) (R&D Systems, USA) for 1 h, followed by incubation with PE-conjugated goat anti-human secondary antibody (1:1000) (Jackson ImmunoResearch, USA). After three washes, the cells were acquired with BD FACS Diva and the data was analyzed with Flow Jo software (version 10.0.6, Tree Star Inc.).

### Fusion and syncytial formation assay

BHK-21 cells were transfected with 0.5 µg of Spike- and/or ACE2- and/or TMPRSS2-expressing plasmids using HD FuGENE transfecting reagent (Promega, E2311) using Opti-MEM media. For the fusion inhibition assay after 2 h post transfection, inhibitors such as E-64d (20 µM, Sigma-Aldrich, E8640), Camostat mesylate (100 U, Sigma-Aldrich, SML0057) and NH_4_Cl (20 mM, Sigma-Aldrich, 254134) were added to the respective wells. For trypsin-dependent cell–cell fusion, after 22 h of transfection, TPCK-trypsin (2 mg/ml) treatment was given for one hour to the respective well in DMEM without FBS. Later, the media was changed with complete DMEM containing 10% FBS. Cells were fixed after 24 h of transfection in 4% paraformaldehyde and then permeabilized with 0.1% Triton in PBS. Nonspecific binding was blocked using 3% goat serum in PBS for 1 h at room temperature. Cells were then incubated overnight at 4 °C with anti-Spike polyclonal sera (1:200). The next day, the cells were washed with PBS and incubated with Alexa 488-labeled rabbit anti-mouse IgG (1:1000). After washing, the cell nuclei were counterstained with DAPI (D9542, Sigma-Aldrich, United States). The expression of proteins was observed by fluorescence microscopy (IX-71, Olympus).

### Flow cytometry based dual dye fusion assay

HEK293T cells were seeded in a 12-well plate and 0.5 µg of Spike- and/or ACE2- and/or TMPRSS2-expressing plasmids were transfected using HD FuGENE transfecting reagent (Promega, E2311) next day. For the fusion inhibition assay after 4 h of transfection, inhibitors such as E-64d (20 µM, Sigma-Aldrich, E8640), Camostat mesylate (100 U, Sigma-Aldrich, SML0057) and NH4Cl (20 mM, Sigma-Aldrich, 254134) were added to the respective wells. For trypsin-dependent cell–cell fusion, after 22 h of transfection, TPCK-trypsin (2 mg/ml) treatment was given for one-hour to the respective well in DMEM without FBS. Later, the media was changed with complete DMEM containing 10% FBS. Cells were stained with CytoPainter cell tracking orange and green dye (abcam, ab138891 and ab138892, respectively) according to the user manual. After staining, spike and hACE2/hACE2 + TMPRSS2 expressing cells were added in equal ratio and incubated for 8 h. Post incubation, cells were washed with FACS buffer (3% FBS in PBS) and fixed with 2% paraformaldehyde. Cells were then acquired on BD FACSCanto II and analyzed on FlowJo.

#### Live virus infection and plaque assay

SARS-CoV-2 Wuhan-1 and variants live virus assays were performed in in THSTI Infectious Disease Research Facility (Biosafety level 3 facility). VeroE6, Caco2, Calu3-hACE2, A549 and BEAS-2b cells were infected with Wuhan-1, Alpha, Delta, and Omicron viruses at 0.1 MOI. After 1 h of virus adsorption, the plates were washed once with plain DMEM. DMEM growth media supplemented with inhibitors (E-64d, 20 µM, Sigma-Aldrich, E8640; Camostat mesylate, 100 U, Sigma-Aldrich, SML0057; trypsin, 2 mg/ml and NH4Cl, 20 mM, Sigma-Aldrich, 254134) was added to the infected wells. Wells with no inhibitors were used as virus control. Post 48 h of infection, cells were either fixed for immunofluorescence assay as explained above to compare spike protein intensities of different viruses or cells lysate and supernatant was harvested for quantitative RT-PCR and plaque assay, respectively.

For live-virus based ELISA assay to check Wuhan-1 and Delta RBD sera cross reactivity with Omicron, VeroE6 cells were seeded and infected in a 96-well plate. Post 24 h of infection, plate was fixed and stained with Wuhan-1 and Delta RBD sera and further stained with goat anti-mouse HRP conjugated secondary antibody. After washing, TMB substrate was added and the reaction was stopped with 2 N H_2_O_4_. Absorbance was measured at 450 nm.

Viral burden was measured by plaque assay in Vero E6 cells. Briefly, 80% confluent Vero E6 cells were infected with serially diluted viruses for 1 h at 37 °C. After 1 h of adsorption, the plate was washed once with plain DMEM and overlaid with DMEM growth medium containing 2% carboxy methylcellulose (CMC) and incubated at 37 °C. Forty-eight hours post infection, the plate was fixed in 4% formalin for an hour and stained with 1% crystal violet for 30 min at RT and washed under running tap water.

### Measurement of viral load

Cell lysate samples were kept in Trizol (Invitrogen) and stored at − 80 °C. RNA was extracted as per the manufacturer’s protocol. Relative copy number estimation of SARS-CoV-2 RNA was performed. Briefly, 1 µg of RNA was reverse transcribed by using the applied biosystem high-capacity cDNA reverse transcription kit. Amplification was accomplished over 40 cycles. Briefly, 150 ng of RNA was used as a template for reverse transcription-polymerase chain reaction (RT-PCR) to target a highly conserved region of the N gene (forward primer: ATGCTGCAATCGTGCTACAA; reverse primer: GACTGCCGCCTCTGCTC). Detection and sub-genomic RNA copy numbers were estimated by the Δ*C*_t_ method. b-actin and GAPDH primers were used as an endogenous control for normalization through quantitative RT-PCR. Primers are as follows: β-actin-For-5′GATGTATGAAGGCTTTGGTC-3′, Rev-5′-TGTGCACTTTTATTGGTCTC-3′; GAPDH-For-5′-ACAGTTGCCATGTAGACC-3′; Rev-5′-TTGAGCACAGGGTACTTTA-3′. Deducted the CT value of the endogenous control gene β-actin or GAPDH from the *C*_T_ value of the N gene to generate the change in the cycling threshold (Δ*C*_T_). The relative expression of the N gene was expressed as “viral load (log10)” relative to that of the respective samples. We used the formula (POWER (2,−Δ*C*_T_)*10,000 to calculate the relative gene expression. The acquisition of qPCR was done on Applied Biosystems 7500 RT-PCR systems and analyzed the data results by using software SDS2.1.

### Statistical analysis

The results were analyzed on GraphPad Prism version 7 (GraphPad Software Inc.). Significant differences were calculated by comparing Wuhan-1 with other variants using *t* test, one- or two-way ANOVA (analysis of variance) with Bonferroni’s multiple comparisons test. Values were considered statistically significant for p values below 0.05. *P* values are presented as asterisks as *P < 0.05, **P < 0.01, ***P < 0.001, ****P < 0.0001 considered significant.

## Supplementary Information

Below is the link to the electronic supplementary material.

Supplementary file1 (DOCX 6099 kb)

## Data Availability

The data supporting the findings of this study are available from the corresponding author upon request.
