# Supplementary material for: Intrinsic D614G and P681R/H mutations in SARS-CoV-2 VoCs Alpha, Delta, Omicron and viruses with D614G plus key signature mutations in spike protein alters fusogenicity and infectivity
Source: Med Microbiol Immunol. 2022 Dec 30;212(1):103–22. doi: 10.1007/s00430-022-00760-7 (PMC9801140; doi:10.1007/s00430-022-00760-7)
Supplement: Supplementary file 1 — Supplementary file1 (DOCX 6099 kb) [file 430_2022_760_MOESM1_ESM.docx]

**Supplementary Information for manuscript titled “**Intrinsic D614G and P681R/H mutations in SARS-CoV-2 VoCs Alpha, Delta, Omicron and viruses with D614G plus key signature mutations in spike protein alter fusogenicity and infectivity”

Ritika Khatri^1^, Gazala Siddqui^1^, Srikanth Sadhu^1, 2^, Vikas Maithil^1^, Preeti Vishwakarma^1^, Bharat Lohiya^1^, Abhishek Goswami^1^, Shubbir Ahmed^1^, Amit Awasthi^1, 2^, Sweety Samal^1#^

Affiliations:

^1^Translational Health Science & Technology Institute, NCR Biotech Science Cluster, Faridabad, Haryana 121001, India.

*Correspondence：Sweety Samal, sweety.samal@thsti.res.in

This file includes:

Figures S1 to S7

Supplementary Figures

**Supplementary figure 1**


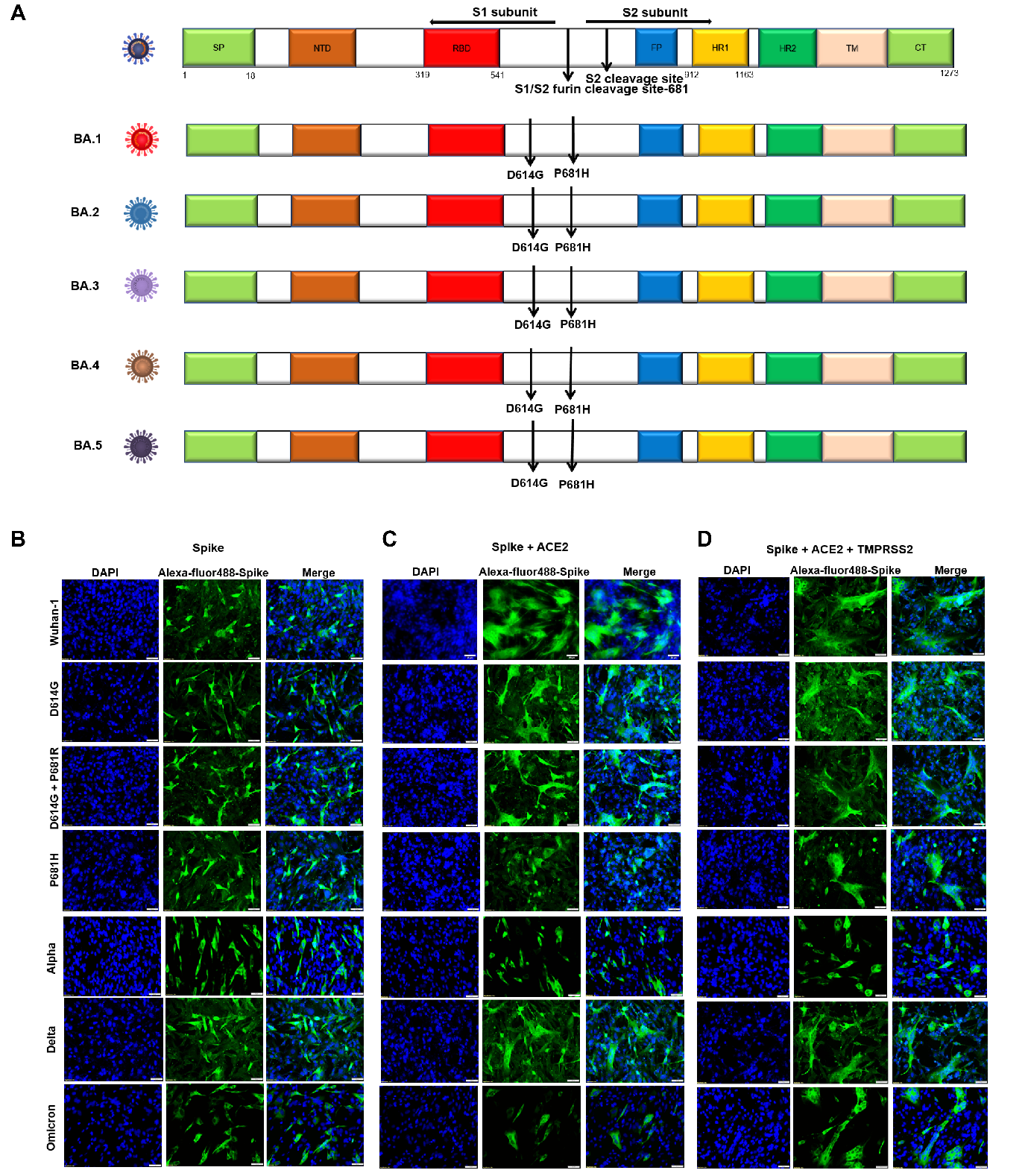


**Figure S1. Omicron variants conserve D614G and P681H mutations and expression of SARS-CoV-2 Wuhan-1 and variants spike protein.** A. Schematic representation of Omicron lineages spike protein showing conserved D614G and P681R mutation. B-D. BHK-21 cells were transfected with spike protein, spike + hACE2 and spike + hACE2 + TMPRSS2 respectively. Spike and hACE2; and spike, hACE2 and TMPRSS2 mediated cell fusion was used as an inhibitor control for the fusion analysis in Fig. 3C and 6D respectively. 24 hours post transfection, the cells were fixed and the expression was checked by probing with primary anti-spike mouse polyclonal sera (1:200) (polyclonal mouse sera raised against Wuhan-1 spike protein, BEI Resources NR-52394) and secondary antibody Alexa-Fluor 488-labeled anti-mouse antibody (green) (1:1000). The images were taken in Olympus fluorescence microscope. The experiments were repeated three times. The nuclei were stained with 4′,6-diamidino-2-phenylindole (DAPI, blue); scale bar: 50 μm and magnification 20x.

**Supplementary figure 2**


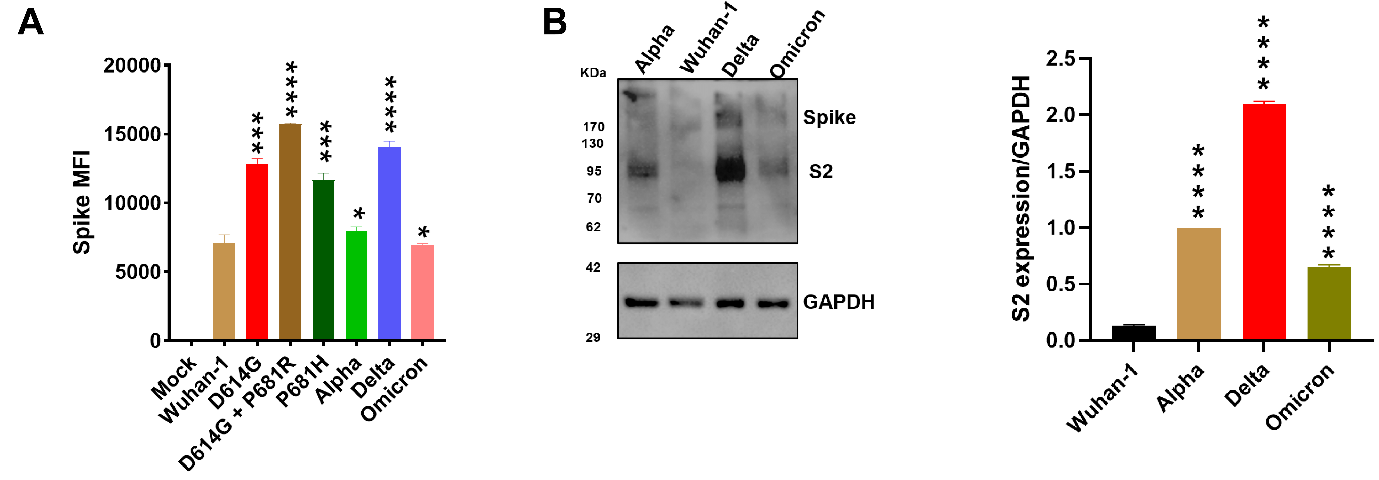


**Figure S2. Spike expression of variants spike protein as measured by flow cytometry and western blot.** A. FACS-based cell surface expression assay was carried out by transfecting the HEK293T cells with different spike plasmids. 24 hours post transfection cells were harvested using 5mM EDTA, washed three times with FACS buffer (PBS+ 3% HIFBS) and stained with anti-spike mouse polyclonal sera for 1 hour at room temperature (RT). The cells were washed three times with FACS buffer and then stained with PE-conjugated goat anti-mouse secondary antibody (1:200 dilutions, Jackson ImmunoResearch) for 1 hour at RT. The cells were again washed three times with FACS buffer and fixed with 0.5% paraformaldehyde. The stained cells were then analyzed in a FACS Canto analyzer (BD Biosciences) and data analyzed with FlowJo software (version 10.0.6, Tree Star Inc). The data were expressed as mean fluorescent intensity (MFI). B. A549 cells were infected with SARS-CoV-2 live viruses at 0.1 MOI in and at 48-hours post infection cell extracts were collected and spike protein was detected by western blot analysis. GAPDH was used as a loading control. Statistical significance was determined using one-way ANOVA, with multiple comparisons test keeping Wuhan-1 as control. Where (p < 0.05), *P <0.05, **P<0.01, ***P<0.001, ****P<0.0001 were considered significant and p>0.05 was considered nonsignificant (ns).

**Supplementary figure 3.**


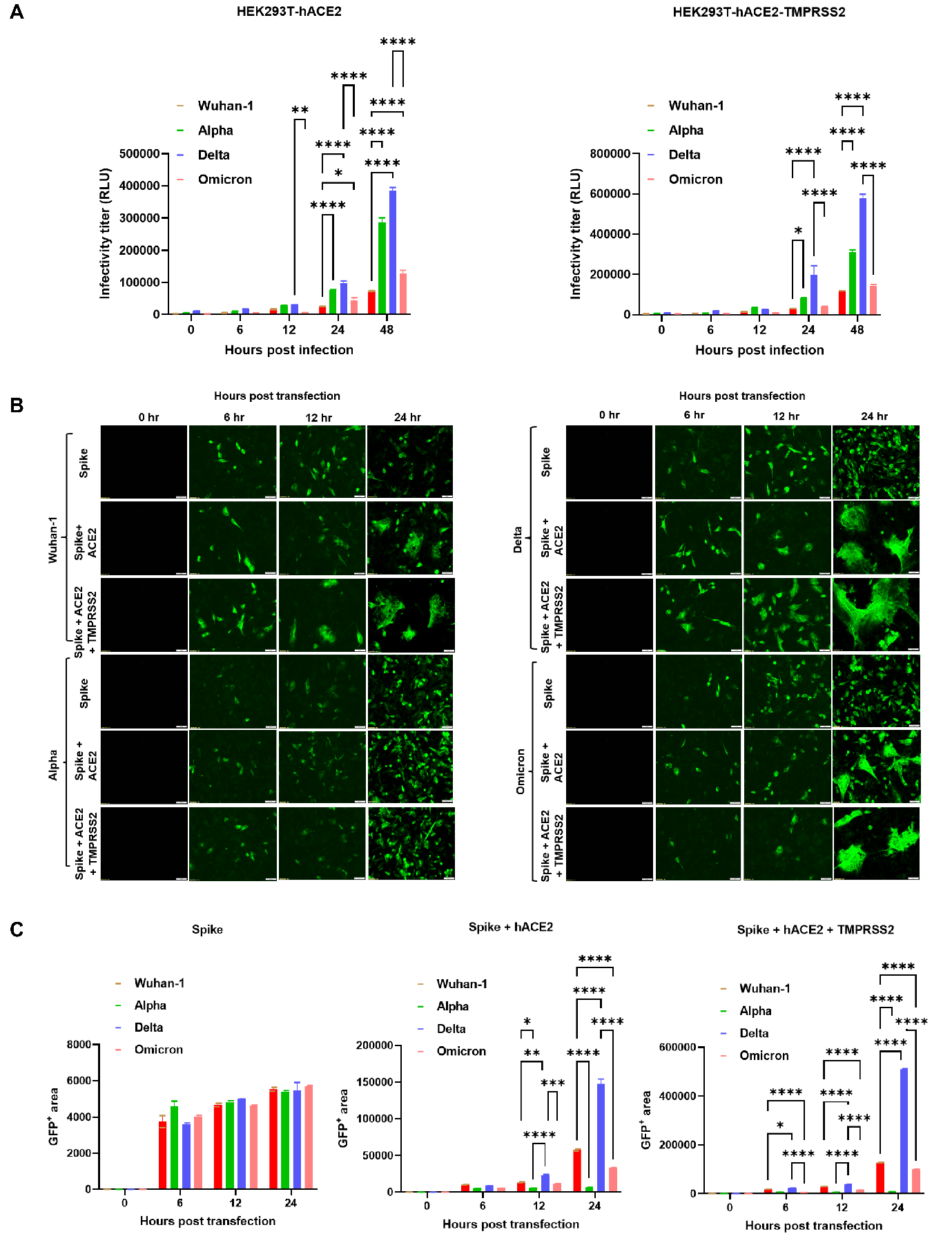


**Figure S3: Time-point based infectivity and fusion assay of Wuhan-1 and VoCs.** A. Wuhan-1, Alpha, Delta and omicron pseudoviruses were produced in HEK293T cells, and infectivity was measured in the HEK293T cells overexpressing hACE2 and HEK293T cells overexpressing hACE2 and TMPRSS2 at 0, 6-, 12-, 24- and 48-hours post infection as relative luciferase units (RLU). The data shown are the averages of three experiments in duplicates. B-C. Wuhan-1 and VoCs spike, spike + hACE2 and spike + hACE2 + TMPRSS2 plasmids were co-transfected into BHK-21 cells and fusion and syncytia formation was measured at 0-, 6-, 12- and 24-hours post transfection. The images were taken with an Olympus fluorescence microscope and quantification was done by comparing three randomly selected GFP+ areas. The experiments were repeated three times. The nuclei were stained with 4′,6-diamidino-2-phenylindole (DAPI, blue); scale bar: 50 μm and magnification 20x. Statistical significance was determined using two-way ANOVA, with multiple comparisons. Where (p < 0.05), *P <0.05, **P<0.01, ***P<0.001, ****P<0.0001 were considered significant and p>0.05 was considered nonsignificant (ns).

**Supplementary figure 4.**


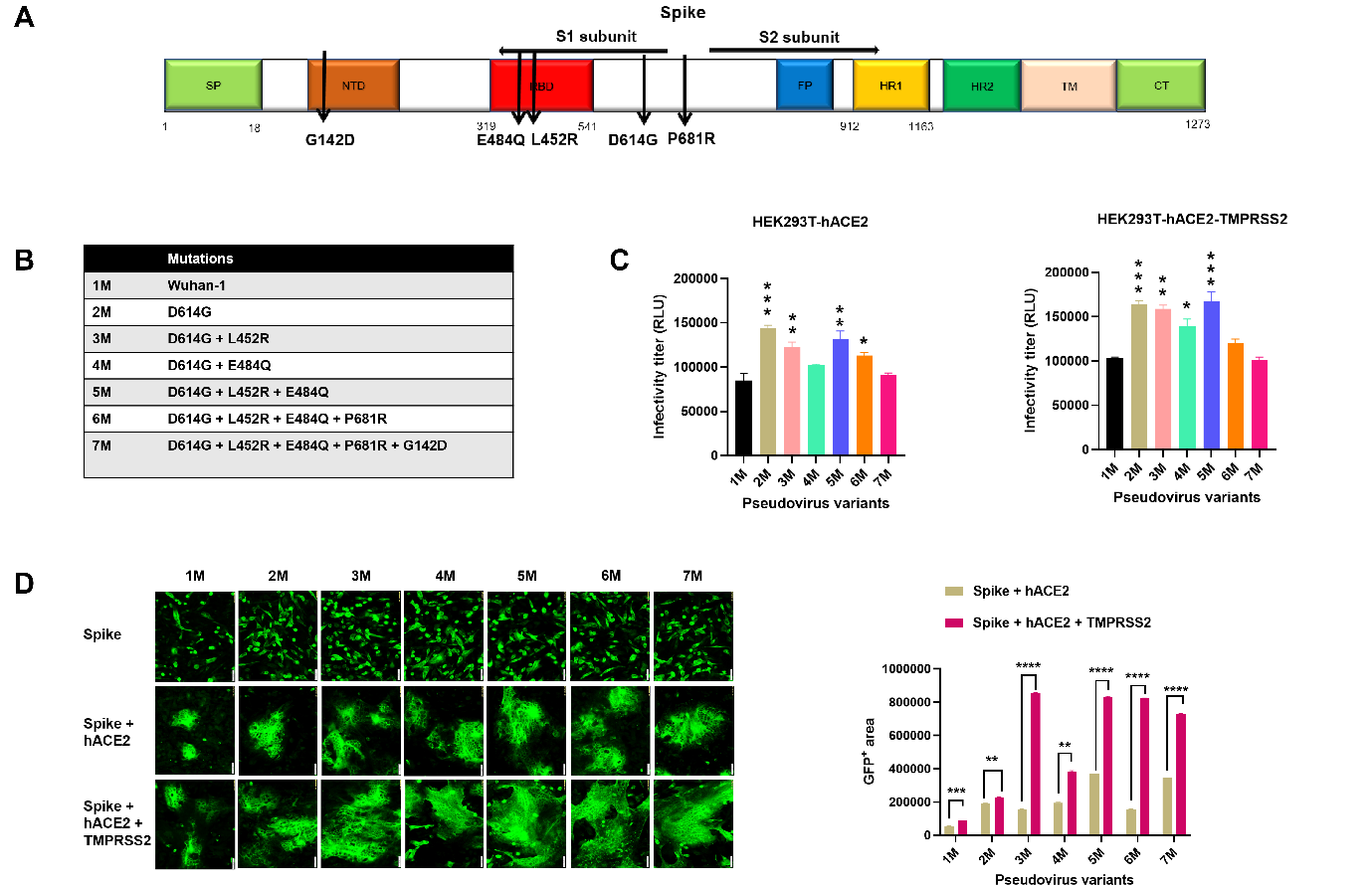


**Figure S4: Infectivity and fusion assay of key selected mutations of VoCs. A**. Graphical representation showing key selected mutations in the spike protein**.** B. Table abbreviating the mutations present in the mutants 1M to 7M. C. Infectivity titer of the mutants 1M-7M in HEK293T cells overexpressing hACE2 and HEK293T cells overexpressing hACE2 and TMPRSS2 at 48-hours post infection as relative luciferase units (RLU). The data shown are the averages of three experiments in duplicates. D. Mutant Spike, spike + hACE2 and spike + hACE2 + TMPRSS2 plasmids were co-transfected into BHK-21 cells and fusion and syncytia formation was measured at 24-hours post transfection. The images were taken with an Olympus fluorescence microscope and quantification was done by comparing three randomly selected GFP+ areas. The experiments were repeated three times. The nuclei were stained with 4′,6-diamidino-2-phenylindole (DAPI, blue); scale bar: 50 μm and magnification 20x. Statistical significance was determined using one way or two-way ANOVA, with multiple comparisons. Where (p < 0.05), *P <0.05, **P<0.01, ***P<0.001, ****P<0.0001 were considered significant and p>0.05 was considered nonsignificant (ns).

**Supplementary figure 5.**


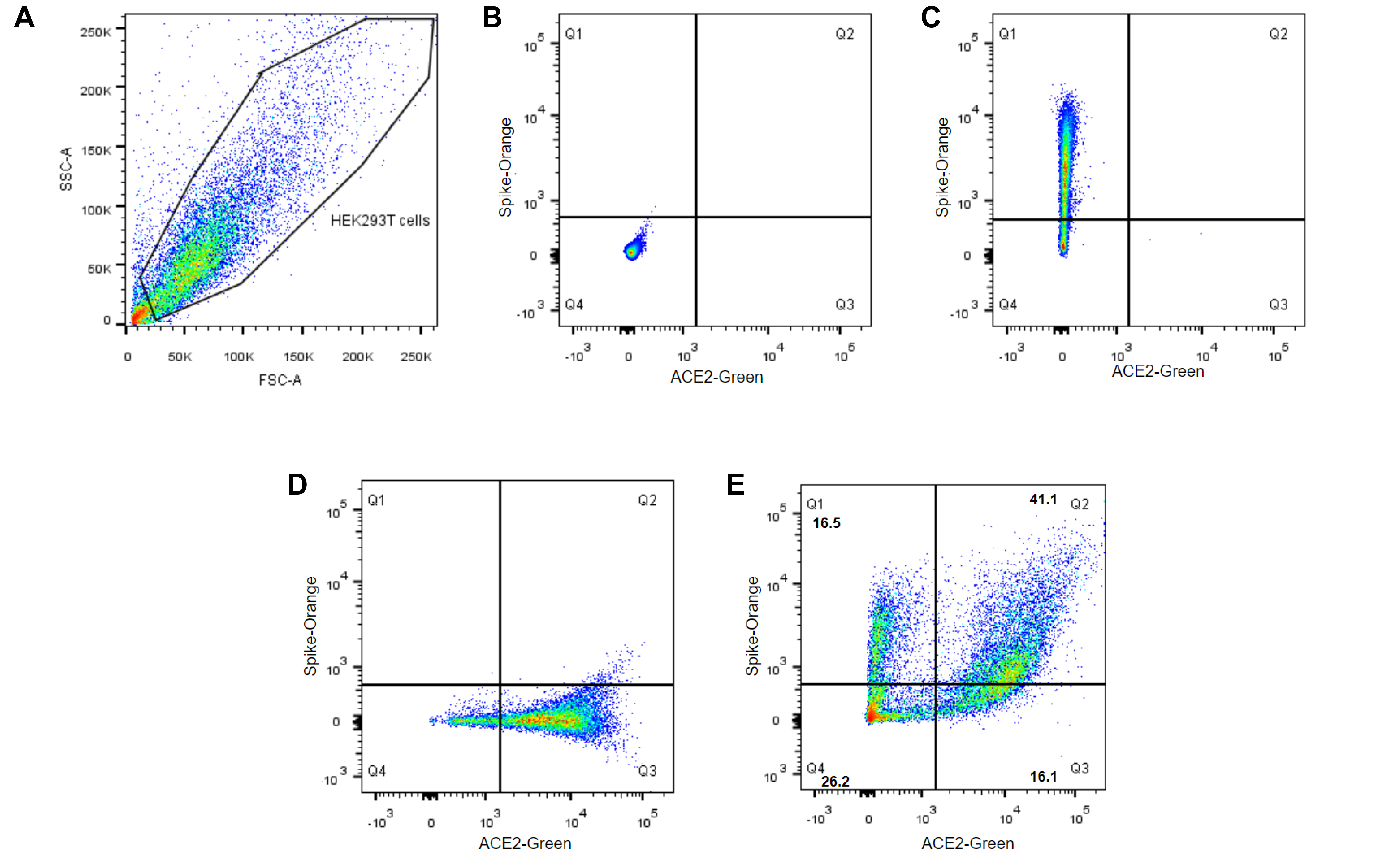


**Figure S5: Gating strategy for flow cytometry-based cell-cell fusion assay.** A. HEK293T cells were gated on the basis of SSC and FSC. B-D. Gates were set based on unstained control, Spike expressing orange dye cell control and ACE2 expressing green dye cell control. E. A positive sample gated on the basis of single dye controls showing frequency distribution for different quadrants.

**Supplementary figure 6.**


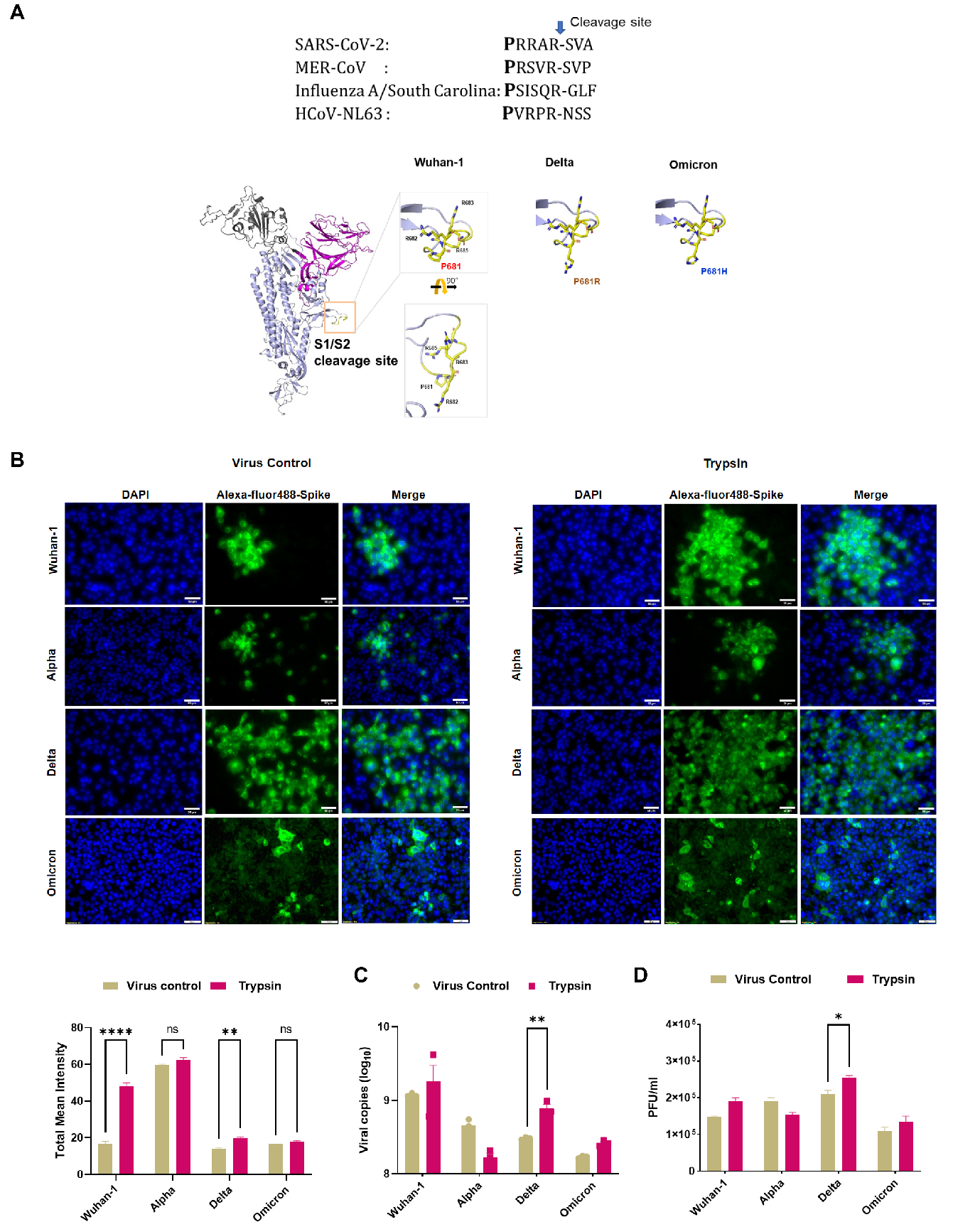


**Figure S6. Effect of trypsin on the live viruses’ replication.** A. Cleavage site sequences and the overall cartoon representation of a monomer of the ectodomain of the SARS-CoV-2 spike glycoprotein in the prefusion conformation. The RBD is shown in gray, the NTD in purple and the S2 domain in light blue. The furin cleavage site at the junction of S1 and S2 is shown in yellow. This region is a flexible loop-like structure and exposed in the prefusion conformation for easy accessibility to proteases. The cleavage site is rich in Arg residues. A magnified view of the cleavage site with the Arg residues shown as sticks. B. VeroE6 cells were infected with Wuhan-1 and VoCs at 0.2 MOI for 48 hours in the absence or presence of trypsin (2ug/ml). Cells were then fixed and probed with anti-spike (Wuhan-1) polyclonal mouse sera (1:200 dilution) and Alexa Fluor 488-labeled anti-mouse secondary antibody (green) (1:1000 dilution). The images were taken with an Olympus fluorescence microscope. The nuclei were stained with DAPI, blue; scale bar: 50 μm and magnification 20x. C-D. VeroE6 cells were seeded and infected as mentioned above. Cell lysates were harvested with trizol post 48 hours of infection and relative copy number of SARS-CoV-2 N gene was estimated by quantitative RT-PCR keeping GAPDH or β-actin genes as an endogenous control for normalization. Cell supernatant was serially diluted and VeroE6 cells were infected to determine plaque forming units (Pfu/ml). Statistical significance was determined using t test and using virus infection as a control.

**Supplementary figure 7.**


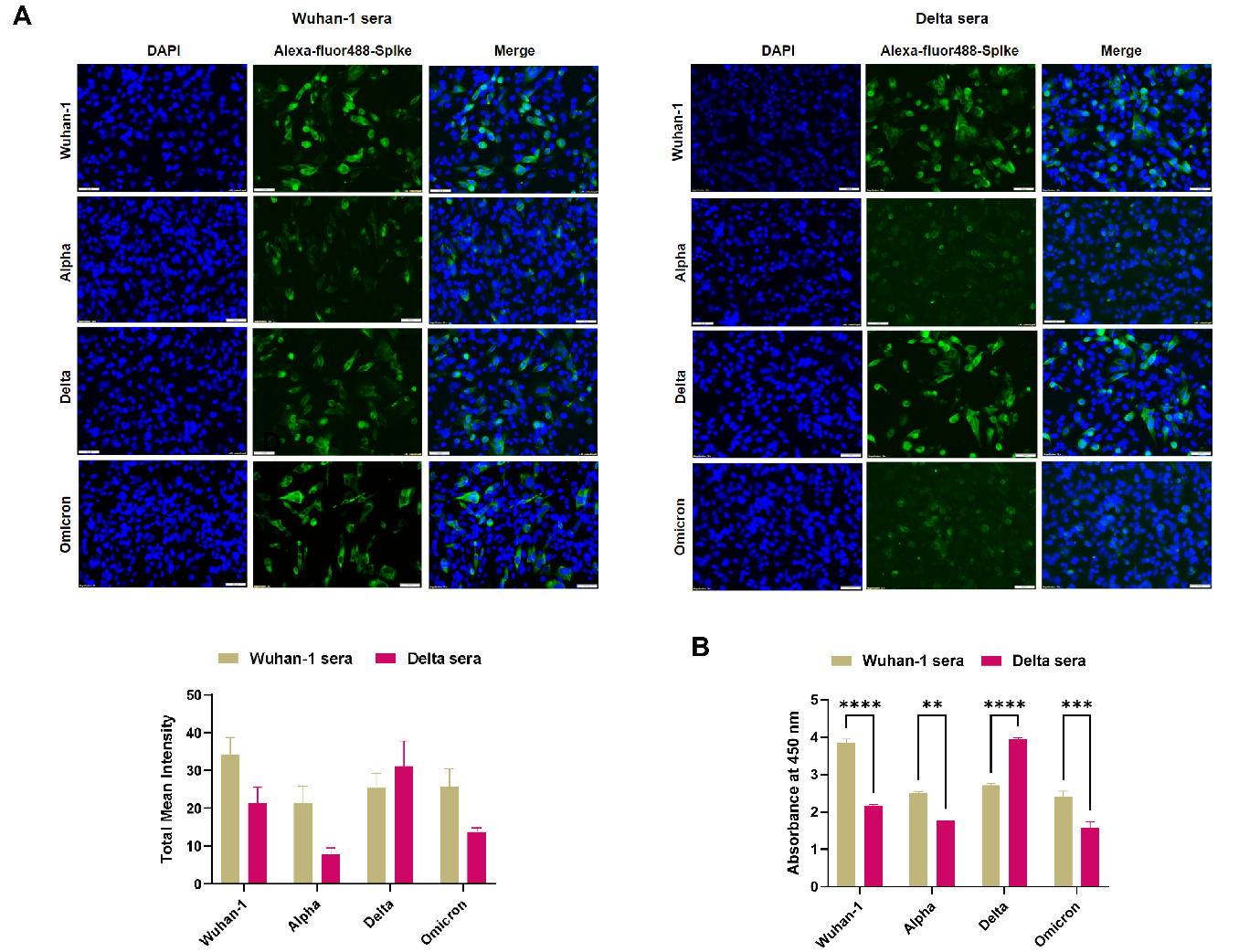


**Figure S7: Cross reactivity of live virus Omicron spike proteins with anti-Wuhan-1 type and anti-Delta RBD polyclonal mouse sera in Vero E6 cells.** A. Briefly, VeroE6 cells infected with Wuhan-1 and VoCs at. 0.1 MOI and 48-hours post infection cells were fixed and probed with anti-Wuahn-1 RBD or anti-Delta RBD polyclonal mouse sera (1:200 dilution) and Alexa Fluor 488-labeled anti-mouse secondary antibody (green) (1:1000 dilution). The images were taken with an Olympus fluorescence microscope. The nuclei were stained with 4′,6-diamidino-2-phenylindole (DAPI, blue); scale bar: 50 μm and magnification 20x.). B. VeroE6 cells were infected and stained as mentioned above. After primary antibody, cells were washed and HRP conjugated anti- mouse secondary antibody was added for 1 hour at room temperature. After washing the plate, TMB substrate was added and the reaction was stopped with 2N H2O4. Absorbance was measured at 450 nm. Statistical significance was determined using two-way ANOVA. *P <0.05, **P<0.01, and ***P<0.001 were considered significant.
